# Thiophene-imidazoline derivatives with varying chain lengths modified by organic acids as corrosion inhibitors for carbon steel in the CO_2_-saturated oilfield produced water

**DOI:** 10.1039/d5ra04201a

**Published:** 2025-09-03

**Authors:** Shuxin Jia, Lei Xiong, Sisi Du, Lin Shen, Yonggang Yu, Jiangbing Li, Zhenglei Wu

**Affiliations:** a School of Chemistry and Chemical Engineering/State Key Laboratory Incubation Base for Green Processing of Chemical Engineering, Shihezi University Shihezi 832003 China jiashuxin2021@163.com 18891556110@163.com dusisi@stu.shzu.edu.cn 18841518147@163.com ljbing@126.com; b Karamay Zhongke Hengxin Technology Co., Ltd Karamay China jingsenyu@126.com; c Xinjiang Jintai Advanced Material Technologies Co., Ltd Huyanghe China 13912944872@139.com

## Abstract

The dissolution of CO_2_ in oilfield produced water causes severe pipeline corrosion and economic losses, highlighting the critical need for medium–high temperature corrosion inhibitors for carbon steel protection. Imidazoline derivative corrosion inhibitors S4-C7 (thiophene-imidazoline octanamide), S4-C9 (thiophene-imidazoline decanamide), S4-C11 (thiophene-imidazoline lauramide) and S4-C13 (thiophene-imidazoline myristamide) with different carbon chain lengths were synthesized by modifying thiophene-imidazoline with different organic acids. At medium–high temperatures, weight loss measurements, electrochemical tests, surface morphology analysis, and theoretical calculations were employed to investigate their inhibition performances and mechanisms in CO_2_-containing solutions. The findings indicate that S4-C11 exhibits excellent corrosion inhibition performance. Specifically, when its concentration reaches 100 mg L^−1^, it can attain an inhibition efficiency of 87.55%. In addition, the investigation was carried out to elucidate the underlying factors contributing to the disparities in the corrosion inhibition efficiencies among corrosion inhibitors with varying carbon chain lengths. One of the reasons is that the carbon chain length affects the hydrophobicity of the corrosion inhibitors. Through quantum chemical calculations and molecular dynamics simulations, it has been firmly established that, in comparison with S4-C7, S4-C11 exhibits a more remarkable electron-donating ability. Moreover, S4-C11 shows lower adsorption energy on carbon steel surfaces and forms a more compact protective film, which collectively contributes to its superior performance. These combined properties more effectively limit contact between the steel surface and corrosive species, thereby inhibiting further corrosion.

## Abstract 2

The study on the effect of imidazoline corrosion inhibitor carbon chain length on carbon steel corrosion inhibition.

## Graphical abstract

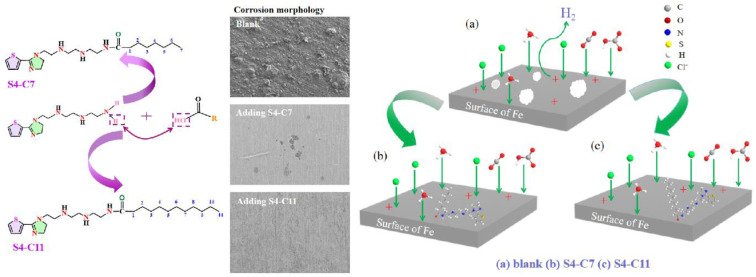

## 1. Introduction

CO_2_ capture, utilization, and storage (CCUS) technology serves as a pivotal technological approach for advancing the green transition in the hydrocarbon industry, demonstrating dual functionality in enabling enhanced oil recovery (EOR) and contributing substantively to carbon peaking and carbon neutrality objectives.^1–4^ However, the dissolution of CO_2_ in produced water from oilfields can induce severe corrosion in well pipelines. CO_2_ corrosion presents a considerable hazard to the safety during the exploitation, transportation, and storage of oil and gas resources. CO_2_ corrosion, also known as sweet corrosion, is a common type of corrosion in the oil and gas industries and is one of the main factors to be considered in the application of carbon.^5–8^ In general, when the pH remains the same, H_2_CO_3_ demonstrates a higher degree of corrosivity compared to HCl or other strong acids.^9,10^ Alternatively, it is of critical necessity to adopt efficient measures for the reduction of steel corrosion in environments containing CO_2_.

Incorporating corrosion inhibitors is a commonly-employed and well-established anticorrosion technique. It allows continuous operation, offers diverse options for different environments and metals, is cost-effective, and doesn't demand high operator skills.^11–13^ Some organic compounds are used to inhibit carbon steel corrosion. S, O, and N, which are heteroatoms in these compounds, interact with metal atoms through bonding, thereby generating protective films that cover the metal surface. Given their low toxicity and eco-friendly characteristics, imidazoline and its derivatives have been extensively utilized to inhibit the corrosion of steels within CO_2_ environments.^14,15^ Nevertheless, imidazoline exhibits certain limitations, including low water solubility and susceptibility to hydrolysis.^14,15^ These characteristics significantly compromise its corrosion inhibition efficiency. With the aim of augmenting the inhibitory capacity of imidazoline, some investigations into the synergistic effects of imidazoline inhibitors in combination with other substances have been carried out.^16–20^ Studies indicate that mixing imidazolines with substances like thiourea can enhance inhibition, but the improvement is limited by the inhibitor's molecular structure. Modifying this structure is a more effective way to boost inhibition performance.

Existing studies have shown that the corrosion inhibitions characteristics of imidazoline-containing inhibitors can be manipulated. This alteration may be realized by means of modifying the lengths of the alkyl chains in imidazoline molecular structures.^21,22^ These research endeavors are mainly concentrated on elucidating the effect of the length of hydrophobic carbon chains on the anti-corrosion efficiency of imidazoline. Most methods for increasing the carbon chain length are achieved by using acids with different carbon chain lengths in the synthesis of imidazoline raw materials.^23–25^ Such understanding focuses on how the molecular structure of imidazoline-type inhibitors is related to their efficiency in corrosion inhibition. This exploration can also provide a theoretical basis for the development of new and highly efficient imidazoline – based inhibitors.

At present, quantum chemical calculations and molecular dynamics simulations are among the most commonly employed theoretical computational methods. Currently, widely used theoretical computational methods include quantum chemical calculations and molecular dynamics simulations.^26–29^ Employing density functional theory (DFT), the research team headed by Zhang^30^ conducted quantum chemical calculations on the inhibitors LDT and S-LDT. The findings showed that the *E*_HOMO_ value of S-LDT is higher than that of LDT, which suggests that S-LDT has a more pronounced electron-donating capacity. Additionally, S-LDT has a smaller energy gap (Δ*E*) than LDT, suggesting enhanced electron transfer between S-LDT and the carbon steel surface, thereby contributing to its superior corrosion inhibition performance. By means of molecular dynamics (MD) simulations, Zhang's research team^31^ determined the diffusion coefficients of corrosive species in not only the aqueous (H_2_O) phase but also the phases of the adsorbed SBLC or TU films. Corrosive species exhibited a much higher diffusion coefficient in the solution without inhibitor compared to that in the film created by the inhibitor. This clearly demonstrates that the corrosion inhibitor effectively prevents corrosive substances from reaching the surface of carbon steel.

In this work, *via* the amidation reaction, imidazoline corrosion inhibitors with different carbon chain lengths (S4-C7 to S4-C13) were prepared by modifying thiophene-imidazoline with different organic acids. Through weight loss measurements, electrochemical methods, and an analysis of surface morphologies before and after corrosion, the corrosion inhibition performance of S4-C7 to S4-C13 on carbon steel in CO_2_-saturated formation water was studied. Moreover, quantum chemical calculations and molecular dynamics simulations were applied to reveal the inhibition mechanisms of the new-type corrosion inhibitors.

## 2. Experimental section

### 2.1 Synthesis of corrosion inhibitors

**Fig. 1:**
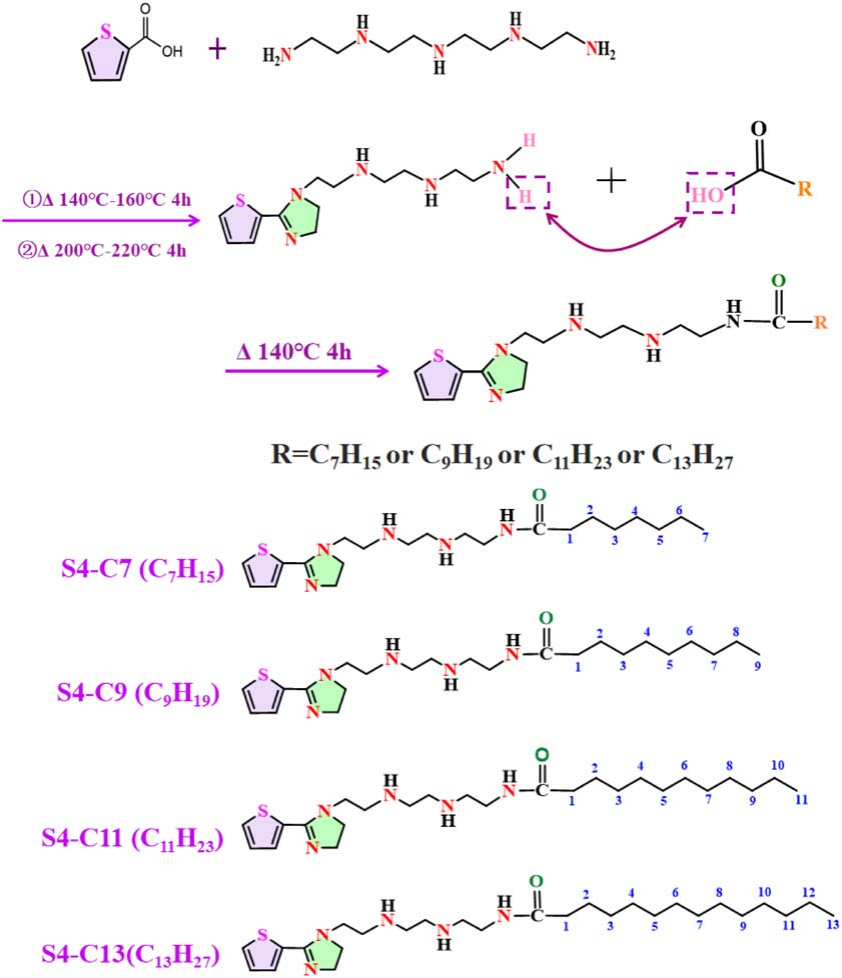
Preparation flow chart of imidazoline corrosion inhibitor.

As illustrated in Fig. 1, four imidazoline derivatives were synthesized, and the detailed synthesis procedures are described below. (i) 0.04 mol 2-thiophenecarboxylic acid, 0.04 mol tetraethylenepentamine and 25 mL dimethylbenzene (solvent) were added into the three-necked flask, (ii) the mixture was heated at 140 °C for 4 h with stirring under N_2_, then was further heated to 220 °C and reacted for 5 h, thiophene-imidazoline S4 was obtained. (iii) Separately, 0.04 mol of each of *n*-caprylic acid, decanoic acid, lauric acid, and myristic acid was introduced into the three – necked flask, and (iv) under N_2_ flow, the mixture was heated to 140 °C and maintained at this temperature for 5 h with continuous stirring. Ultimately, brown viscous liquids were obtained and named S4-C7, S4-C9, S4-C11 and S4-C13 respectively.

### 2.2 Molecular structure characterization

By means of a Fourier transform infrared spectrometer, the molecular structure was characterized. The synthesized inhibitors' ^1^H NMR spectra were examined *via* nuclear magnetic resonance spectroscopy.

### 2.3 Material and test solution

Q235 carbon steel was selected as the subject material. Its chemical composition, in weight percentage (%), was as follows: 0.30Mn, 0.22C, 0.35Si, 0.045S, 0.045P, with the remainder being Fe. The test solution, composed of 3.5wt% NaCl, 0.06wt% CaCl_2_, 0.03wt% NaHCO_3_, 0.02wt% MgCl_2_, was prepared from analytical reagents to simulate formation water. The pH of the CO_2_-saturated formation water was 4.13.

### 2.4 Weight loss measurements

Q235 carbon steel samples were fabricated with the dimensions of 50 mm in length, 13 mm in width, and 1.5 mm in thickness. Subsequently, all these specimens were immersed in CO_2_-saturated formation water containing different inhibitor concentrations and maintained at 90 °C for a duration of 22 hours. Prior to conducting the experiment, the samples underwent a series of pretreatment steps: they were cleaned with absolute ethanol, dried under a continuous flow of N_2_ gas, and precisely weighed using an analytical balance. To ensure the reliability and reproducibility of the experimental data, three specimens were simultaneously tested for each set of experimental conditions. Following the immersion process, the corrosion products on the specimen surface were removed by applying a de-filming solution prepared with 6 g of hexamethylenetetramine, 100 mL of hydrochloric acid, and 900 mL of water. Subsequently, the corrosion rate (*ν*, in units of mm/a) was determined using eqn (1).1
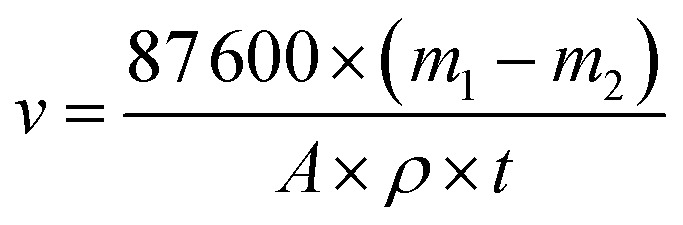
where *m*_1_ and *m*_2_ represent the weights before and after the experiment, respectively (g), *A* represents the surface area (cm^2^), *ρ* represents the density of the specimen (g cm^−3^), and *t* represents the experiment duration (h). The inhibition efficiency (*η*, %) can be calculated using eqn (2):2
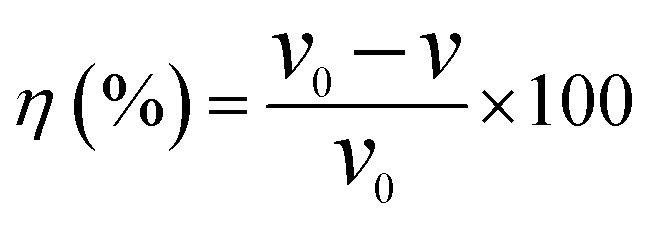
where *ν*_0_ and *ν*_1_ represent the corrosion rates of the samples in the solution without or with inhibitor, respectively.

### 2.5 Electrochemical measurements

Carbon steel samples with the dimension of 10 mm × 10 mm × 1 mm were used in this work. The samples were sealed with epoxy with the exposed area of 1.0 cm^2^. For electrochemical measurements, a copper wire was soldered to each sample for electric connection. Before corrosion tests, the carbon steel samples were abraded with a series of SiC papers (800#, 1500#, 2000# and 2500#).

All of the electrochemical tests were conducted in a three-electrode cell (including a working electrode (carbon steel sample), reference electrode (a saturated calomel electrode (SCE)), and counter electrode (a platinum sheet)). Before the tests, CO_2_ gas was purged into the test solution for 1 h for deoxygenation, and then the sample was immersed in the solution quickly, and CO_2_ gas was continuously purged into the test solution to keep a CO_2_-saturated condition during the whole test. After the sample was immersed in the solution for 60 min to obtain a steady open circuit potential (OCP), electrochemical impedance spectroscopy (EIS) tests were performed at OCP in the frequency from 100 000 to 0.1 Hz with 5 mV of sinusoidal perturbation. Finally, polarisation curves (PC) were measured from −0.25 V to 0.25 V *vs.* OCP with a sweeping rate of 0.5 mV s^−1^.

### 2.6 Surface analyses

All specimens were exposed to CO_2_-saturated formation water with or without 100 mg per L inhibitors for 22 h at 90 °C. This inhibitor concentration was chosen according to preliminary electrochemical and weight loss measurements, which indicated that it demonstrated the best inhibition effect. The surface morphology of the specimens was observed by scanning electronic microscopy (SEM). The contact angle (CA) of water drops on the specimens was measured at 25 °C using a CA goniometer. The X-ray photoelectron spectroscopy (XPS) was employed to measure the surface composition of the samples. The surface roughness of the samples is measured using an atomic force microscope (AFM).

### 2.7 Quantum chemical calculations

Based on density functional theory (DFT) and the B3LYP/6-311G(d, p) method,^30^ quantum chemical calculations are employed to determine the optimized structures of S4-C7 and S4-C11 while considering the solvent model (water). Gaussian 16 W software is used to carry out these calculations. The results are post-processed and visualized by Multiwfn^32^ and Visual Molecular Dynamic (VMD) software.^33^

### 2.8 Diffusion of corrosive species by molecular dynamics (MD) simulations

The inhibition effect of the adsorbed S4-C7 and S4-C11 films was explored by calculating the diffusion process of corrosive species (such as Cl^−^, H_3_O^+^, H_2_O, CO_2_, HCO_3_^−^, H_2_CO_3_) in the blank solution (water phase) and the adsorbed S4-C7 or S4-C11 films through MD simulations.^31^ Firstly, a three-dimensional diffusion model was constructed. For the system without inhibitor, a H_2_O box containing 500H_2_O, 5H_3_O^+^, 5HCO_3_^−^, 5H_2_CO_3_, 5CO_2_, 5Cl^−^, and 5Na^+^ was built. For the adsorbed S4-C7 or S4-C11 film system, a S4-C7 box or S4-C11 box including 200 S4-C7 or S4-C11 molecules, 5H_2_O, 5H_3_O^+^, 5HCO_3_^−^, 5H_2_CO_3_, 5CO_2_, 5Cl^−^, and 5Na^+^ was constructed. Then, the geometry of each system was optimized. Finally, MD simulations were performed for the diffusion model of each system under the isothermal isobaric (*NPT*) ensemble for 200 ps and then under the canonical ensemble (*NVT*) for 500 ps at 363.15 K by applying the COMPASS II force field. After MD simulations, the diffusion coefficient (*D*) of each species was determined by the mean square displacement (MSD).

## 3. Results and discussion

### 3.1 Characterization of imidazoline derivatives

The infrared (IR) spectra of four imidazoline derivatives and imidazoline S4 are shown in Fig. S1. In the IR spectrum of S4, the absorption peak at 3300 cm^−1^ corresponds to the N–H stretching vibration of the –NH_2_ group, confirming that S4 is a monocyclic imidazoline. For S4-C11, the strong and broad peak observed at approximately 3318 cm^−1^ is attributed to the N–H stretching vibration of the –NH group, while the peak at 1381 cm^−1^ arises from the C=O stretching vibration. These two characteristic peaks collectively indicate the successful amidation reaction. Additionally, the absorption bands at 1632 cm^−1^ and 2884 cm^−1^ are assigned to the C=N^34^ and C–N stretching vibrations, respectively, further confirming the presence of the imidazoline ring. A comparison of the IR spectra of S4-C7, S4-C9, S4-C13, and S4-C11 reveals their structural similarity. For instance, characteristic peaks corresponding to the –NH group (3318 cm^−1^), CH_2_ asymmetric stretching in the alkyl chain (2924 cm^−1^), CH_3_ deformation vibration (2971 cm^−1^), and C–S stretching vibration (1051 cm^−1^) are consistently observed in all four derivatives. These IR spectroscopic results provide clear evidence for the successful synthesis of the target compounds.

The structures of the four imidazoline derivatives were further characterized by ^1^H NMR spectroscopy, as shown in Fig. S2. The successful acid modification was confirmed by the presence of characteristic peaks at chemical shifts of approximately 1.59 and 2.22 ppm, along with the integration value of protons at around 1.25 ppm. For instance, in the ^1^H NMR spectrum of S4-C11, the proton integration at *δ* 1.25 ppm corresponds to 16 protons, providing clear evidence for the successful synthesis of S4-C11.

For S4-C7, ^1^H NMR (400 MHz, CDCl_3_): *δ* 7.49 (t, *J* = 3.4 Hz, 1H, S–CH̲–CH), 7.46–7.44 (m, 1H, C–CH̲–CH), 7.06 (s, 1H, CH–CH̲–CH), 3.79 (s, 2H, N–CH̲_2_–CH_2_), 2.58 (t, *J* = 5.3 Hz, 4H, (NH–CH̲_2_–CH_2_)_2_), 2.49 (s, 4H, NH–CH̲_2_–CH̲_2_–NH), 2.16 (t, *J* = 6.4 Hz, 2H, O=C–CH̲_2_–CH_2_), 1.59 (d, *J* = 7.3 Hz, 2H, O=C–CH_2_–CH̲_2_), 1.27 (d, *J* = 2.2 Hz, 8H, (CH̲_2_)_4_), 0.87 (d, *J* = 2.0 Hz, 5H, CH̲_3_, (C–NH̲–C)_2_).

For S4-C9, ^1^H NMR (400 MHz, CDCl_3_): *δ* 7.43 (d, *J* = 4.5 Hz, 1H, S–CH̲–CH), 7.32 (d, *J* = 4.2 Hz, 1H, C–CH̲–CH), 7.09–7.02 (m, 1H, CH–CH̲–CH), 3.90–3.66 (m, 2H, N–CH̲_2_–CH_2_), 3.55–3.36 (m, 4H, CH_2_–CH̲_2_–N, N–CH̲_2_–CH_2_), 2.48 (s, 4H, NH–CH̲_2_–CH̲_2_–NH), 2.22 (t, *J* = 7.6 Hz, 2H, O=C–CH̲_2_–CH_2_), 1.58 (t, *J* = 7.4 Hz, 2H, O=C–CH_2_–CH̲_2_), 1.24 (s, 12H, (CH̲_2_)_6_), 0.86 (t, *J* = 6.8 Hz, 5H, CH̲_3_, (C–NH̲–C)_2_).

For S4-C11, ^1^H NMR (400 MHz, CDCl_3_): ^1^H NMR (400 MHz, CDCl_3_) *δ* 7.45 (s, 1H, S–CH̲–CH), 7.04 (s, 1H, C–CH̲–CH), 3.71 (q, *J* = 7.0 Hz, 4H, CH_2_–CH̲_2_–N=, CH_2_–CH̲_2_–N), 3.47 (s, 2H, CH_2_–CH̲_2_–N), 2.28 (t, *J* = 7.6 Hz, 2H, O=C–CH̲_2_–CH_2_), 1.59 (t, *J* = 7.4 Hz, 2H, O=C–CH_2_–CH̲_2_), 1.25 (s, 16H, (CH̲_2_)_8_), 0.90–0.84 (m, 5H, CH̲_3_, (C–NH̲–C)_2_).

For S4-C13, ^1^H NMR (400 MHz, CDCl_3_): ^1^H NMR (400 MHz, CDCl_3_) *δ* 7.45 (s, 1H, S–CH̲–CH), 7.38 (d, *J* = 4.8 Hz, 1H, C–CH̲–CH), 7.06 (s, 1H, CH–CH̲–CH), 3.77 (s, 4H, CH_2_–CH̲_2_–N=, CH_2_–CH̲_2_–N), 3.46 (s, 4H, CH_2_–CH̲_2_–N, N–CH̲_2_–CH_2_), 2.94–2.68 (m, 4H, (NH–CH̲_2_–CH_2_)_2_), 2.47 (s, 4H, NH–CH̲_2_–CH̲_2_–NH), 2.24–2.13 (m, 2H, O=C–CH̲_2_–CH_2_), 1.59 (t, *J* = 7.3 Hz, 2H, O=C–CH_2_–CH̲_2_), 1.24 (s, 20H, (CH̲_2_)_10_), 0.87 (t, *J* = 6.8 Hz, 5H, CH̲_3_, (C–NH̲–C)_2_).

**Fig. 2:**
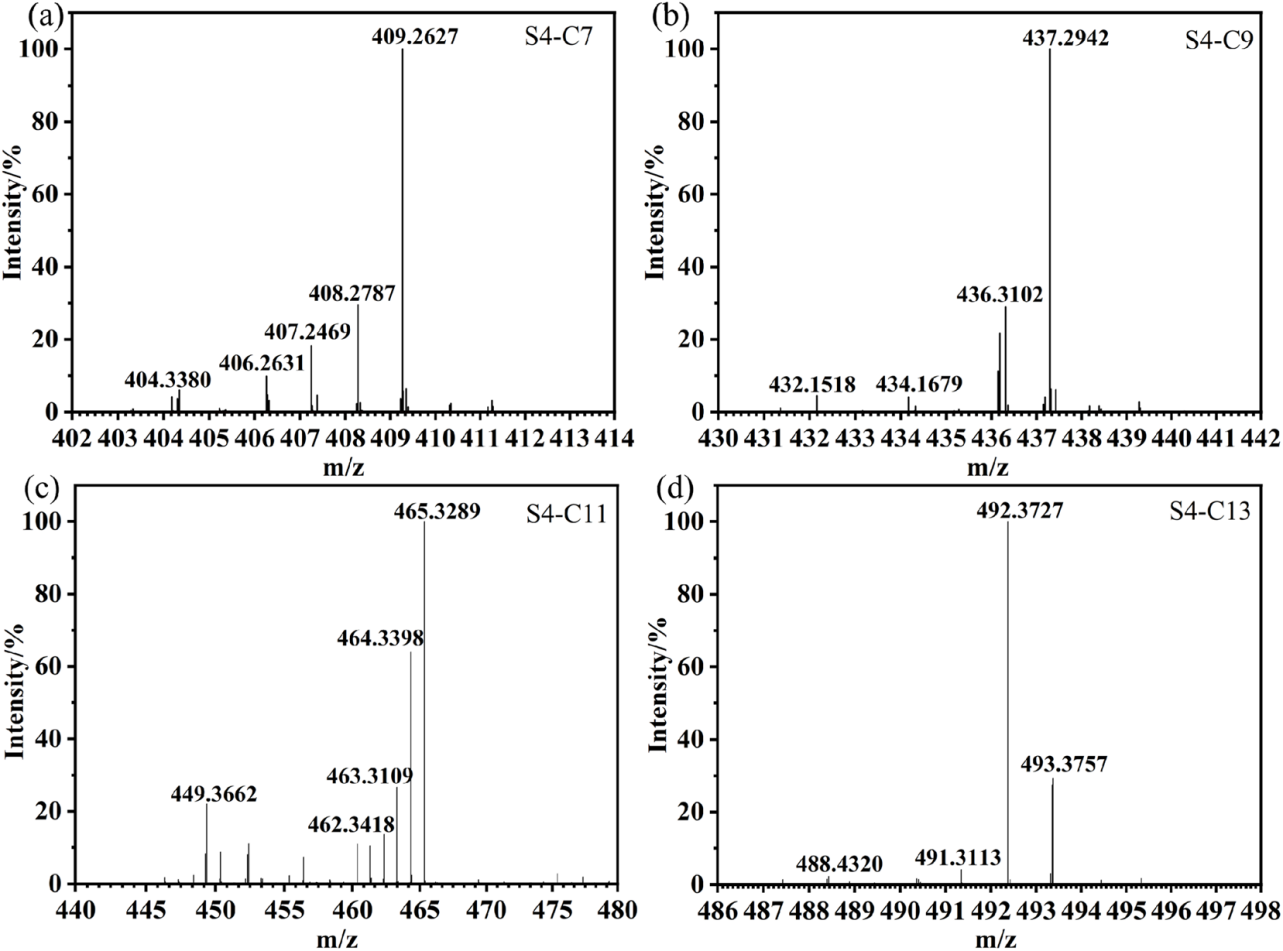
High resolution mass spectrum of imidazoline derivatives (a) S4-C7; (b) S4-C9; (c) S4-C11; (d) S4-C13.

In addition, we carried out mass spectrometry, and obtained data display in Fig. 2. Mass spectrometry analysis confirmed the successful synthesis of the target corrosion inhibitor molecules S4-C7, S4-C9, S4-C11, and S4-C13. The observed [M + H]^+^ peaks were in excellent agreement with their theoretical molecular weights: 407.2719 (C_21_H_37_N_5_OS) for S4-C7, 435.3032 (C_23_H_41_N_5_OS) for S4-C9, 463.3345 (C_25_H_45_N_5_OS) for S4-C11, and 491.3658 (C_27_H_49_N_5_OS) for S4-C13. These mass spectrometric results, combined with the infrared spectroscopy and ^1^H NMR data, provide conclusive evidence for the successful synthesis of all four imidazoline derivatives.

### 3.2 Weight loss measurements

**Fig. 3:**
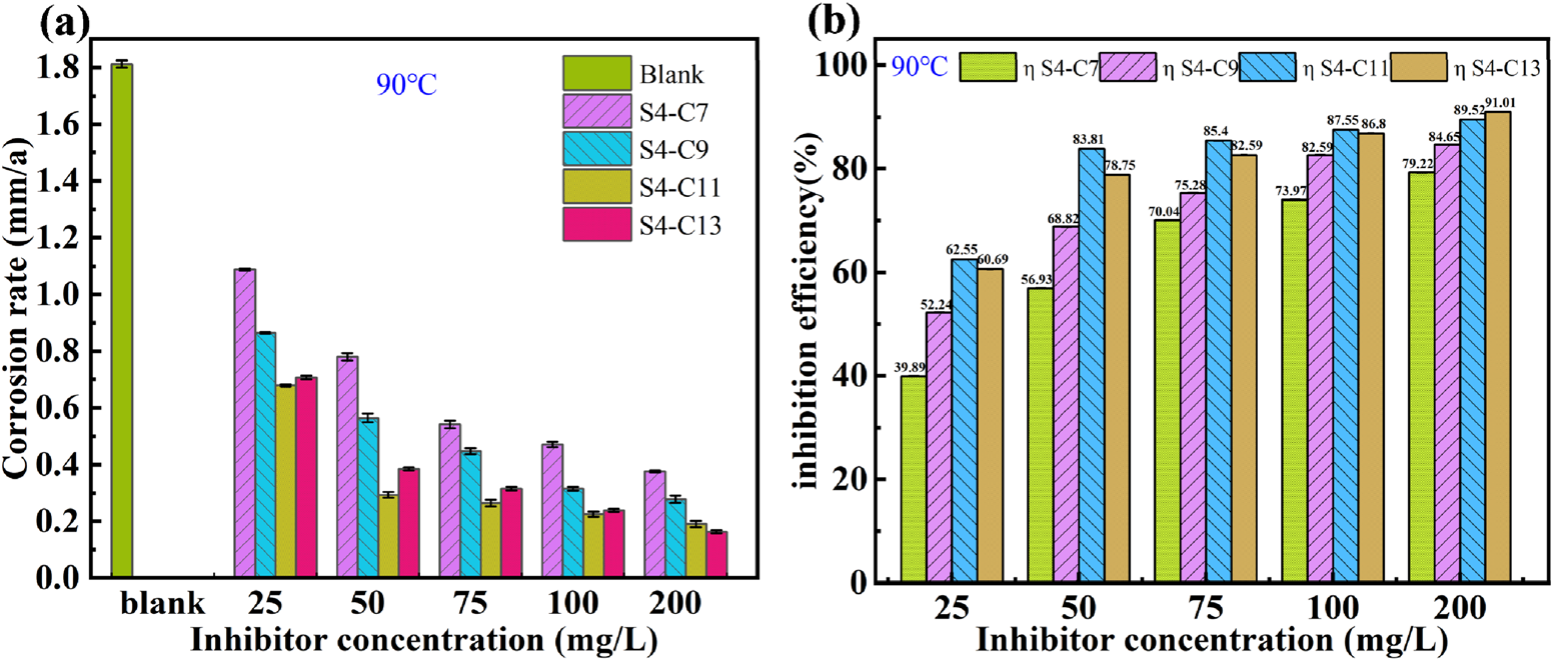
Corrosion inhibition efficiency and carbon steel corrosion rate at different inhibitor concentrations in CO_2_ systems.

The corrosion rates of carbon steel in blank solution and in solutions containing different concentrations of imidazoline derivatives, as well as the corresponding inhibition efficiency of the inhibitors, are shown in Fig. 3. Table 1 presents the corresponding mass loss results.

**Table 1:** 90 °C Weight loss experimental data with varying concentrations of corrosion inhibitor

*c* (mg L^−1^)	Inhibitor	*m*_1_ (g)	*m*_2_ (g)	Δ*m* (g)	*v* (mm/a)	Average *v* (mm/a)	*η*	Average *η*
25	S4-C7	7.5852	7.5530	0.0322	1.0923	1.0889	39.70%	39.89%
		7.4512	7.4192	0.0320	1.0855		40.08%	
	S4-C9	7.6202	7.5945	0.0257	0.8702	0.8652	51.96%	52.24%
		7.5263	7.5009	0.0254	0.8602		52.51%	
	S4-C11	7.6315	7.6118	0.0197	0.6683	0.6785	63.11%	62.55%
		7.6284	7.6081	0.0203	0.6886		61.99%	
	S4-C13	7.3584	7.3377	0.0207	0.7022	0.7073	61.24%	60.96%
		7.5687	7.5477	0.0210	0.7124		60.67%	
50	S4-C7	7.5138	7.4904	0.0234	0.7938	0.7803	56.18%	56.93%
		7.6549	7.6323	0.0226	0.7667		57.68%	
	S4-C9	7.2716	7.2554	0.0162	0.5495	0.5648	69.66%	68.82%
		7.4579	7.4408	0.0171	0.5801		67.98%	
	S4-C11	7.4249	7.4166	0.0083	0.2816	0.2935	84.46%	83.81%
		7.6147	7.6057	0.0090	0.3053		83.15%	
	S4-C13	7.5214	7.5098	0.0116	0.3935	0.3850	78.28%	78.75%
		7.6103	7.5902	0.0111	0.3765		79.21%	
75	S4-C7	7.4757	7.4593	0.0164	0.5563	0.5428	69.29%	70.04%
		7.7514	7.7358	0.0156	0.5292		70.79%	
	S4-C9	7.4633	7.4498	0.0135	0.4580	0.4478	74.72%	75.28%
		7.4561	7.4432	0.0129	0.4376		75.84%	
	S4-C11	7.3577	7.3498	0.0079	0.2680	0.2646	85.21%	85.40%
		7.3782	7.3705	0.0077	0.2612		85.58%	
	S4-C13	7.5290	7.5194	0.0096	0.3257	0.3155	82.02%	82.59%
		7.4675	7.4585	0.0090	0.3053		83.15%	
100	S4-C7	7.4245	7.4109	0.0136	0.4613	0.4715	74.53%	73.97%
		7.5431	7.5289	0.0142	0.4817		73.41%	
	S4-C9	7.5357	7.5263	0.0094	0.3189	0.3155	82.40%	82.59%
		7.5175	7.5083	0.0092	0.3121		82.77%	
	S4-C11	7.3155	7.3085	0.0070	0.2375	0.2256	86.89%	87.55%
		7.3541	7.3478	0.0063	0.2137		88.20%	
	S4-C13	7.2602	7.2535	0.0067	0.2273	0.2392	87.45%	86.80%
		7.4268	7.4194	0.0074	0.2510		86.14%	
200	S4-C7	7.4977	7.4867	0.0110	0.3731	0.3765	79.40%	79.22%
		7.5721	7.5609	0.0112	0.3799		79.03%	
	S4-C9	7.4646	7.4565	0.0081	0.2748	0.2782	84.83%	84.65%
		7.4384	7.4301	0.0083	0.2816		84.46%	
	S4-C11	7.5005	7.4947	0.0058	0.1968	0.1900	89.14%	89.52%
		7.5955	7.5901	0.0054	0.1832		89.89%	
200	S4-C13	7.5469	7.5420	0.0049	0.1662	0.1628	90.82%	91.01%
		7.6136	7.6089	0.0047	0.1594		91.20%	

It can be observed that at 90 °C, the corrosion rate of carbon steel in the inhibitor-free solution reaches 1.8115 mm/a, indicating extremely severe corrosion. However, in the presence of imidazoline derivative inhibitors, the corrosion rate is significantly reduced. For instance, with the addition of S4-C11 at 100 mg L^−1^ and 200 mg L^−1^, the corrosion rates decrease to 0.2256 mm/a and 0.0215 mm/a, respectively, with corresponding inhibition efficiencies of 87.55% and 89.52%. According to the Petroleum and Natural Gas Industry Standard SY/T 5273-2014, these corrosion rates meet the acceptable criteria. However, further increasing the concentration from 100 mg L^−1^ to 200 mg L^−1^ has a relatively minor effect on reducing the corrosion rate.

At the same concentration, the inhibition efficiency generally follows the order S4-C11 > S4-C13 > S4-C9 > S4-C7. This trend can be attributed to the following reasons:

As the hydrophobic chain length increases, the interaction between inhibitor molecules and water weakens, while hydrophobicity is significantly enhanced, facilitating the formation of a stable protective film on the carbon steel surface, thereby improving inhibition efficiency. However, an excessively long carbon chain may reduce the solubility of the inhibitor, decreasing the number of freely mobile inhibitor molecules per unit volume and hindering their adsorption on the metal surface. This could explain why S4-C13 exhibits slightly lower inhibition efficiency than S4-C11.

### 3.3 Open circuit potential and EIS measurements

**Fig. 4:**
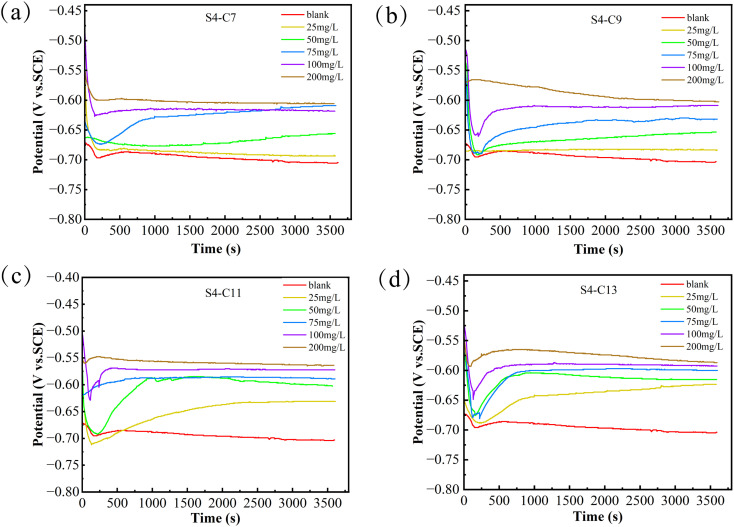
Open-circuit potential variation of carbon steel immersed in simulated oilfield produced water containing carbon dioxide (over 3600 seconds) (a) S4-C7, (b) S4-C9, (c) S4-C11, (d) S4-C13.

Fig. 4 shows the variation of the open-circuit potential (*E*_OCP_) of carbon steel in CO_2_-simulated oilfield produced water at 90 °C, either without or with different concentrations of corrosion inhibitors, as a function of immersion time. It can be observed that after 3600 s of immersion, the *E*_OCP_ of the carbon steel electrode stabilizes, indicating that a relatively steady state has been attained. Compared to the inhibitor-free solution, the open-circuit potential (*E*_OCP_) of carbon steel in the inhibitor-containing solution exhibits a positive shift, which is attributed to the adsorption of inhibitor molecules on the carbon steel surface. As the inhibitor concentration increases, the magnitude of the *E*_OCP_ positive shift becomes more pronounced. For imidazoline amides with different carbon chain lengths, the extent of the positive shift varies even at the same concentration. Based on the experimental results, the four inhibitors can be ranked in descending order of *E*_OCP_ positive shift as follows: S4-C11 > S4-C13 > S4-C9 > S4-C7.

**Fig. 5:**
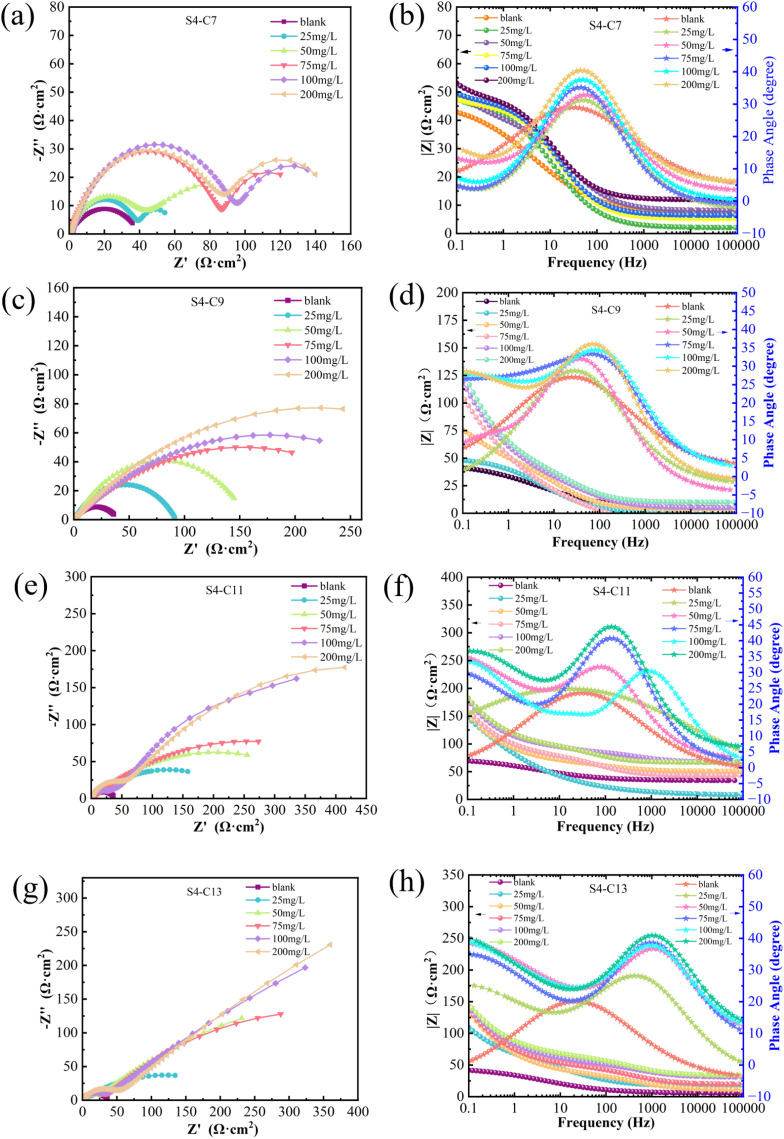
Electrochemical impedance spectroscopy (EIS) of carbon steel in solutions containing different concentrations of corrosion inhibitor (a, c, e and g) Nyquist diagram (b, d, f and h) Bode diagram.

Fig. 5 presents the electrochemical impedance spectra of carbon steel in simulated oilfield produced water without and with corrosion inhibitors. The Nyquist plots exhibit distinct shapes due to factors including elevated test temperature, slight variations in inhibitor properties, and surface heterogeneity of the carbon steel specimens. Notably, the Nyquist plots demonstrate that the diameter of the impedance semicircle significantly increases upon inhibitor addition compared to the blank solution, indicating substantial inhibition of corrosion reactions. Furthermore, this semicircle diameter exhibits a concentration-dependent enhancement with increasing inhibitor dosage. In the Bode plot analysis, systems containing inhibitors consistently display broad phase angle peaks, suggesting the presence of two time constants in the electrode process – a characteristic not observed in the blank solution. Moreover, the magnitude of impedance modulus in Bode plots shows a progressive increase with higher inhibitor concentrations, consistently exceeding the values obtained for the blank solution.

**Fig. 6:**
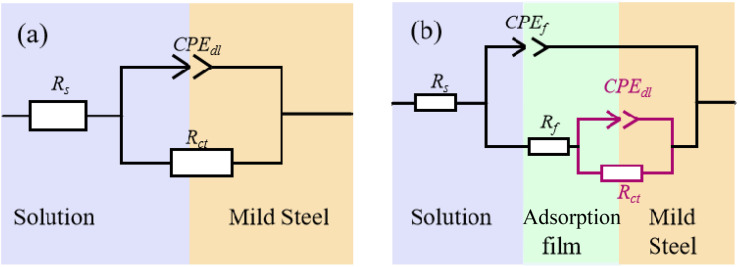
Equivalent circuits for EIS fitting: (a) Blank (b) Containing corrosion inhibitors S4-C7, S4-C9, S4-C11, S4-C13.

The electrochemical impedance spectroscopy data were fitted using the equivalent circuit models *R*_s_(CPE_dl_*R*_ct_) and *R*_s_(CPE_dl_(*R*_ct_(CPE_f_*R*_f_))) shown in Fig. 6, with the corresponding electrochemical parameters obtained from the fitting procedure summarized in Table 2. As illustrated in Fig. 6, the equivalent circuit comprises *R*_s_ (solution resistance), *R*_ct_ (charge transfer resistance), *R*_f_ (inhibitor film resistance), CPE_dl_ (constant phase element representing the double layer capacitance), and CPE_f_ (capacitance of the inhibitor film). For EIS parameter fitting, the double layer capacitance (CPE_dl_) can be substituted by a constant phase element (CPE), whose impedance (*Z*_CPE_) is expressed as:

3 *Z*_CPE_ = *Y*_0_^−1^(*jω*)^−*n*^

where *Y*_0_ represents the CPE magnitude, *j* denotes the imaginary unit, *ω* is the angular frequency, and *n* is the phase shift parameter (0 ≤ *n* ≤ 1) that quantifies surface heterogeneity. When *n* = 0, the CPE behaves as a pure resistor; when *n* = 1, it corresponds to an ideal capacitor. The polarization resistance (*R*_p_) can be calculated from *R*_ct_ and *R*_f_ as follows:For inhibitor-containing systems: *R*_p_ = *R*_ct_ + *R*_f_ (Fig. 6(b))For blank solutions: *R*_p_ = *R*_ct_ (Fig. 6(a))

**Table 2:** Electrochemical impedance spectroscopy (EIS) fitting data of carbon steel in CO_2_ simulated produced water containing varying concentrations of imidazoline derivative inhibitors

Inhibitor	*c* (mg L^−1^)	*R*_s_ (Ω cm^2^)	CPE_f_		*R*_f_ (Ω cm^2^)	CPE_dl_		*R*_ct_ (Ω cm^2^)	*R*_p_ (Ω cm^2^)	*η* (%)
			*Y*_0_ (μΩ^−1^ S^*n*^ cm^−2^)	*n*_f_		*Y*_0_ (μΩ^−1^ S^*n*^ cm^−2^)	*n*_dl_			
Blank	0	5.01 ± 0.03	—	—	—	5010 ± 12.55	0.52 ± 0.03	36.79 ± 8.03	36.79 ± 8.03	—
S4-C7	25	5.56 ± 0.01	2751 ± 13.10	0.87 ± 0.03	44.46 ± 8.03	1443 ± 17.10	0.69 ± 0.03	48.79 ± 9.27	54.35 ± 9.27	33.76
	50	5.47 ± 0.02	2524 ± 12.59	0.85 ± 0.02	50.53 ± 7.06	1111 ± 16.27	0.70 ± 0.053	70.27 ± 8.56	75.74 ± 8.56	52.47
	75	5.11 ± 0.02	2437 ± 11.46	0.82 ± 0.01	56.62 ± 6.05	1105 ± 17.27	0.71 ± 0.043	114.45 ± 7.733	119.56 ± 7.733	69.89
	100	5.27 ± 0.03	2388 ± 12.46	0.81 ± 0.04	59.21 ± 5.02	1152 ± 18.27	0.73 ± 0.053	130.22 ± 8.56	135.49 ± 8.56	73.43
	200	5.46 ± 0.01	2452 ± 11.78	0.81 ± 0.03	59.22 ± 4.55	1416 ± 19.10	0.71 ± 0.073	134.45 ± 8.33	139.91 ± 8.33	74.27
S4-C9	25	5.52 ± 0.04	3636 ± 10.80	0.69 ± 0.04	41.31 ± 5.92	1059 ± 13.71	0.62 ± 0.063	85.50 ± 8.62	91.02 ± 8.62	60.45
	50	5.28 ± 0.03	3312 ± 10.78	0.74 ± 0.01	44.35 ± 5.76	1125 ± 16.37	0.75 ± 0.03	119.37 ± 7.72	124.65 ± 7.72	71.12
	75	5.53 ± 0.06	3657 ± 12.78	0.69 ± 0.02	45.37 ± 4.67	1872 ± 16.51	0.79 ± 0.03	163.72 ± 8.71	169.25 ± 8.71	78.73
	100	5.74 ± 0.07	3642 ± 11.32	0.72 ± 0.04	46.92 ± 3.76	1645 ± 15.61	0.77 ± 0.05	216.21 ± 8.66	221.95 ± 8.66	83.78
	200	5.75 ± 0.08	3325 ± 13.56	0.73 ± 0.06	43.61 ± 7.55	1324 ± 14.31	0.713 ± 0.07	237.16 ± 8.85	242.91 ± 8.85	85.18
S4-C11	25	5.53 ± 0.02	4319 ± 10.89	0.71 ± 0.06	41.11 ± 3.76	1668 ± 13.27	0.53 ± 0.08	116.98 ± 8.36	122.51 ± 8.36	70.20
	50	5.24 ± 0.03	4238 ± 12.37	0.74 ± 0.03	45.54 ± 4.69	1648 ± 11.33	0.49 ± 0.09	250.72 ± 8.44	255.96 ± 8.44	85.88
	75	5.34 ± 0.06	3565 ± 13.45	0.78 ± 0.02	44.52 ± 4.67	1669 ± 17.89	0.53 ± 0.02	268.22 ± 8.36	273.56 ± 8.36	86.84
	100	5.27 ± 0.01	3037 ± 13.67	0.77 ± 0.03	45.63 ± 5.37	1782 ± 19.91	0.57 ± 0.03	409.00 ± 8.31	414.27 ± 8.31	91.31
	200	5.61 ± 0.07	3876 ± 14.67	0.80 ± 0.02	49.99 ± 6.37	1753 ± 15.63	0.55 ± 0.04	427.19 ± 7.639	434.78 ± 7.63	91.72
S4-C13	25	5.52 ± 0.02	2861 ± 11.37	0.63 ± 0.01	44.13 ± 5.31	1871 ± 14.63	0.48 ± 0.06	95.38 ± 7.85	100.90 ± 7.85	64.32
	50	5.13 ± 0.04	2411 ± 11.92	0.72 ± 0.04	42.66 ± 7.71	1841 ± 15.62	0.51 ± 0.05	178.64 ± 7.77	183.77 ± 7.77	80.41
	75	5.69 ± 0.02	2352 ± 14.37	0.72 ± 0.05	45.49 ± 6.61	1935 ± 14.62	0.59 ± 0.05	225.97 ± 6.92	231.66 ± 6.92	84.46
	100	4.99 ± 0.03	2467 ± 13.67	0.71 ± 0.05	41.63 ± 5.37	1912 ± 15.62	0.54 ± 0.05	330.52 ± 7.53	335.51 ± 7.53	89.27
	200	4.87 ± 0.04	2668 ± 14.68	0.71 ± 0.01	48.73 ± 3.36	1997 ± 14.11	0.57 ± 0.06	317.7	322.57	88.85

The inhibition efficiency (*η*) can then be determined using eqn (4).4
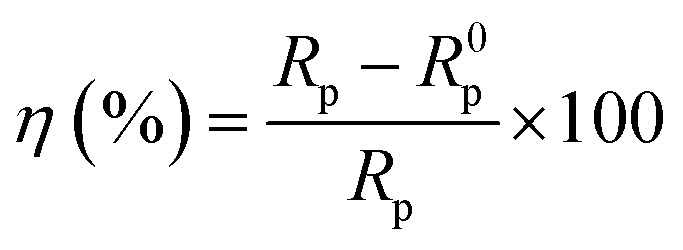


Fig. 5(a) displays the Nyquist plots obtained with varying concentrations of S4-C7, all exhibiting two distinct semicircles. A clear trend is observed where increasing inhibitor concentration leads to enlargement of the semicircle diameters. The appearance of dual semicircles can be mechanistically explained by several factors associated with the high-temperature conditions (90 °C): enhanced corrosion rates may cause partial breakdown of the inhibitor protective film; dynamic equilibrium between adsorption and desorption of inhibitor molecules at the metal surface; development of interfacial heterogeneity that manifests as two separate time constants in the impedance response.

Fig. 5(c) and (e) present the Nyquist plots for systems containing varying concentrations of S4-C9 and S4-C11, respectively. The single semicircle observed in each case indicates that the corrosion process is primarily controlled by charge transfer, suggesting that: the inhibitor films effectively suppress direct corrosion of the carbon steel substrate; the protective films exhibit minimal structural defects. Furthermore, the semicircle radius demonstrates a concentration-dependent increase, consistent with the expected enhancement of protective performance at higher inhibitor concentrations.

Fig. 5(d) shows the Nyquist plots with different concentrations of S4-C13. At relatively low concentrations (below 75 mg L^−1^), a distinct large semicircle with significant curvature appears in the low-frequency region. However, at higher concentrations (100 mg L^−1^ and 200 mg L^−1^), the semicircle in the high-frequency region becomes very small while the slope of the straight line in the low-frequency region increases considerably. This phenomenon can be attributed to the formation of a relatively stable and compact protective film of corrosion inhibitor on the carbon steel surface, which subsequently causes the corrosion process to be predominantly controlled by the diffusion step.

The polarization resistance (*R*^0^_p_) of the blank solution at 90 °C was measured to be 36.79 Ω cm^2^. Higher *R*_p_ values obtained after inhibitor addition indicate superior corrosion inhibition efficiency. Comparative analysis of the *R*_p_ values in Table 2 reveals that at 200 mg L^−1^ concentration, S4-C11 demonstrated the highest polarization resistance (434.78 Ω cm^2^) among the four imidazoline derivatives tested, significantly exceeding the values obtained for S4-C7 (139.91 Ω cm^2^), S4-C9 (242.91 Ω cm^2^), and S4-C13 (322.57 Ω cm^2^). Correspondingly, S4-C11 exhibited the highest inhibition efficiency (91.72%), markedly superior to the other derivatives: S4-C7 (74.27%), S4-C9 (85.18%), and S4-C13 (88.85%).

A comparison of inhibition efficiencies at identical concentrations revealed the following consistent trend: S4-C11 > S4-C13 > S4-C9 > S4-C7, which agrees well with the weight loss measurements. This correlation demonstrates that an optimal increase in carbon chain length enhances polarization resistance by effectively impeding charge transfer processes at the metal–solution interface, thereby improving the corrosion inhibition performance of imidazoline derivatives. However, excessive chain length results in slightly diminished protective efficacy, suggesting the existence of an optimal molecular structure for maximum inhibition performance.

### 3.4 Results of polarization curve measurements

**Fig. 7:**
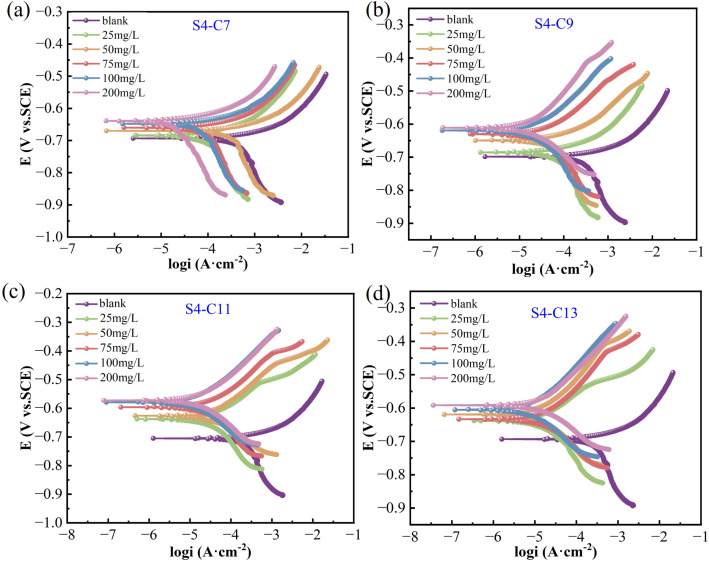
Polarization curves of 80 °C carbon steel with different concentrations of corrosion inhibitors (a) S4-C7 (b) S4-C9 (c) S4-C11 (d) S4-C13.

The corrosion behavior of carbon steel in solutions containing varying concentrations of imidazoline derivatives was investigated through polarization curve measurements, as illustrated in Fig. 7. The electrochemical parameters, including corrosion potential and corrosion current density, were determined using Tafel extrapolation method, with the fitted parameters summarized in Table 3.

**Table 3:** 990 °C in a solution of different concentrations of imidazoline derivatives polarization curve fitting data of carbon steel

Inhibitor	*c* (mg L^−1^)	*E*_corr_ (V *vs.* SCE)	*i*_corr_ (A cm^−2^)	*β*_a_ (mV dec^−1^)	*β*_c_ (mV dec^−1^)	*η* (%)
Blank	Blank	−0.7333	2.8151 × 10^−4^	65.90	−143.18	—
S4-C7	25	−0.7321	1.5801 × 10^−4^	35.45	−115.62	43.87
	50	−0.7016	1.1255 × 10^−4^	33.21	−95.37	60.02
	75	−0.7019	7.2292 × 10^−5^	31.58	−89.31	74.32
	100	−0.6916	6.6690 × 10^−5^	31.21	−88.61	76.31
	200	−0.6901	4.8166 × 10^−5^	30.09	−87.63	82.89
S4-C9	25	−0.7222	1.0407 × 10^−4^	34.61	−103.64	63.03
	50	−0.7183	5.6358 × 10^−5^	30.61	−96.42	79.98
	75	−0.7001	4.1495 × 10^−5^	29.38	−90.57	85.26
	100	−0.6872	3.5667 × 10^−5^	28.34	−86.31	87.33
	200	−0.6775	3.2880 × 10^−5^	27.33	−71.39	88.32
S4-C11	25	−0.6921	3.3331 × 10^−5^	35.61	−95.72	88.16
	50	−0.6831	2.8348 × 10^−5^	31.62	−89.19	89.93
	75	−0.6693	2.6068 × 10^−5^	30.81	−74.62	90.74
	100	−0.6652	2.1479 × 10^−5^	29.81	−64.77	92.37
	200	−0.6645	1.7228 × 10^−5^	27.61	−62.24	93.88
S4-C13	25	−0.6887	3.5695 × 10^−5^	34.62	−88.29	87.32
	50	−0.6821	3.0853 × 10^−5^	32.61	−84.57	89.04
	75	−0.6753	2.8348 × 10^−5^	31.75	−72.19	89.93
	100	−0.6735	2.6068 × 10^−5^	30.61	−69.82	90.74
	200	−0.6639	2.2830 × 10^−5^	28.94	−65.92	91.89

Comparative analysis with the blank solution clearly demonstrates that the addition of all four imidazoline derivatives induces a marked leftward shift of the polarization curves toward lower current density regions, accompanied by significant suppression of both anodic and cathodic current densities, thereby confirming their effective dual inhibition capability on both the anodic metal dissolution reaction and cathodic hydrogen evolution reaction. The observed positive shift in corrosion potential (*E*_corr_) relative to the blank solution, with Δ*E*_corr_ < 85 mV, further confirms that the imidazoline derivatives predominantly inhibit the anodic process. Based on the combined analysis of current density variations and potential shift data, the modified imidazoline derivatives can be classified as anodically-dominated mixed-type corrosion inhibitors.^35^

Comparative analysis of the four imidazoline derivatives' effects on polarization curve displacement reveals that S4-C11 induces the most significant shift (demonstrating both the largest positive potential displacement and greatest movement toward lower current densities), suggesting its superior corrosion inhibition performance. Conversely, S4-C7 exhibits the minimal curve displacement, indicating its relatively poor protective capability among the tested compounds. The addition of corrosion inhibitors significantly reduces the corrosion current density, and the corresponding inhibition efficiency can be quantitatively calculated from the current density values using eqn (5).5
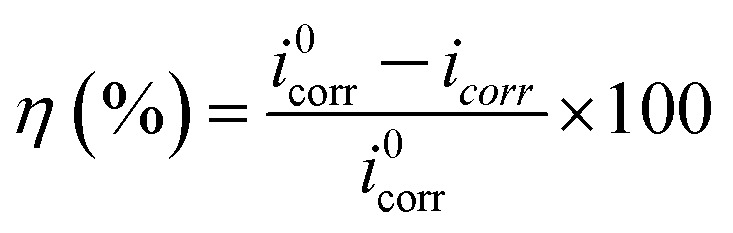
where *i*^0^_corr_ represents the corrosion current density without inhibitor, and *i*_corr_ denotes the corrosion current density measured with inhibitor addition.

The corrosion current density in the blank solution was measured as 2.8151 × 10^−4^ A cm^−2^, while the addition of 100 mg per L imidazoline derivatives significantly reduced the values to 6.6690 × 10^−5^ A cm^−2^ (S4-C7), 3.5667 × 10^−5^ A cm^−2^ (S4-C9), 2.1479 × 10^−5^ A cm^−2^ (S4-C11), 2.6068 × 10^−5^ A cm^−2^ (S4-C13), with corresponding inhibition efficiencies of 76.31% (S4-C7), 87.33% (S4-C9), 92.37% (S4-C11), and 90.74% (S4-C13), demonstrating a consistent performance ranking of S4-C11 > S4-C13 > S4-C9 > S4-C7 that was maintained across all tested concentrations and corroborated by weight loss measurements, open-circuit potential results, and electrochemical impedance spectroscopy data.

### 3.5 Adsorption isotherm

Adsorption isotherms provide critical information regarding the inhibitory adsorption behavior on carbon steel surfaces, including the adsorption–desorption equilibrium constant (*K*_ads_) and standard Gibbs free energy of adsorption (Δ*G*^0^_ads_), thereby enabling quantitative evaluation of the inhibitors' adsorption capacity.^36^ The adsorption characteristics of S4 inhibitors on carbon steel were systematically investigated using appropriate adsorption isotherm models, with the Langmuir adsorption model demonstrating the best fitting performance:6
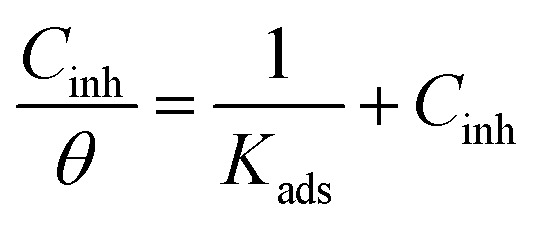
Here, *θ* represents the surface coverage (numerically equivalent to the inhibition efficiency *η*), *C*_inh_ denotes the inhibitor concentration, and *K*_ads_ is the adsorption equilibrium constant. The linear relationships observed in the *C*/*θ versus C* plots (Fig. S3) confirm that the adsorption of imidazoline derivatives follows the Langmuir adsorption isotherm model. Δ*G*^0^_ads_ can be calculated using *K*_ads_, the universal gas constant (*R*), and the absolute temperature (*T*):

7 Δ*G*^0^_ads_ = −*RT* ln(1 × 10^6^*K*_ads_)

The thermodynamic parameters for the adsorption of corrosion inhibitors on carbon steel surfaces are summarized in Table S1.

As evidenced in the aforementioned table, the adsorption equilibrium constant (*K*_ads_) exhibits a progressive decrease with rising temperature, indicating that elevated temperatures adversely affect the adsorption of corrosion inhibitors on carbon steel surfaces. This phenomenon can be attributed to enhanced molecular motion at higher temperatures, which destabilizes chemical bonding and compromises adsorption stability-even if some adsorption occurs at elevated temperatures, the adsorbed molecules become more prone to desorption. At identical temperatures, comparative analysis of *K*_ads_ values for the four imidazoline derivatives reveals the following consistent trend: S4-C11 > S4-C13 > S4-C9 > S4-C7, which correlates well with the performance ranking obtained from experimental testing.

The calculated Gibbs free energy values (Δ*G*^0^_ads_) were all negative, indicating that the adsorption processes of all four imidazoline derivatives on carbon steel surfaces were spontaneous and could achieve corresponding corrosion inhibition effects without requiring specific conditions. Furthermore, the absolute values of Δ*G*^0^_ads_ all exceeded 30 kJ mol^−1^ (*e.g.*, Δ*G*^0^_ads_ = −34.82 kJ mol^−1^ for S4-C11 at 90 °C), demonstrating that the adsorption of these imidazoline derivatives on carbon steel involves both physical and chemical adsorption mechanisms.^37^

### 3.6 Influence of temperature

The adsorption enthalpy (Δ*H*^0^_ads_) can also serve as an indicator for evaluating the adsorption capability of corrosion inhibitors. As shown in eqn (8), a linear relationship between ln(*K*_ads_) and 1/*T* can be established:8
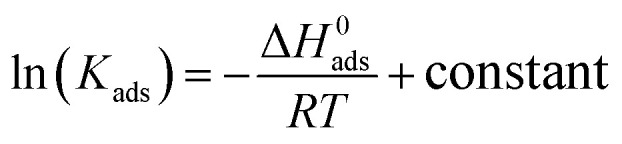


The standard adsorption enthalpy (Δ*H*^0^_ads_) of inhibitor molecules on carbon steel surfaces was determined from the slope of the ln(*K*_ads_) *versus* 1/*T* plot (Fig. S4). The fitting results yielded the following values: Δ*H*^0^_ads_ (S4-C7) = −37.57 kJ mol^−1^, Δ*H*^0^_ads_ (S4-C9) = −31.64 kJ mol^−1^, Δ*H*^0^_ads_ (S4-C11) = −47.42 kJ mol^−1^, and Δ*H*^0^_ads_ (S4-C13) = −45.64 kJ mol^−1^. According to established thermodynamic criteria, when Δ*H*^0^_ads_ values fall within the range of −41.86 to −100 kJ mol^−1^, the adsorption process involves both physical and chemical interactions.^38^ Notably, both S4-C11 and S4-C13 exhibited Δ*H*^0^_ads_ values within this characteristic range (−47.42 and −45.64 kJ mol^−1^, respectively), demonstrating that their adsorption mechanisms on carbon steel surfaces comprise combined physical adsorption and chemical bonding.

The present work systematically investigated the temperature dependence of corrosion rates for carbon steel in the presence of four imidazoline derivatives. The relationship between corrosion rate and temperature was found to obey the Arrhenius equation, which can be linearly transformed into the form shown in eqn (9).9
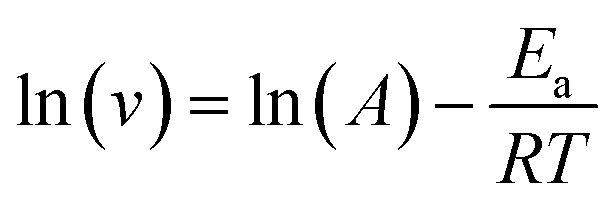


By plotting ln(*v*) *versus* 1/*T* (Fig. S5) and performing linear regression analysis, the relevant parameters for all four inhibitors at 75 mg L^−1^ concentration were obtained. The slopes of the fitted lines were used to determine the activation energy (*E*_a_) for the corrosion process, while the corrosion rate data were derived from weight loss measurements. The complete set of derived parameters is presented in Table S2.

As clearly demonstrated by the experimental results, the absolute value of the slope of the ln(*v*) *versus* 1/*T* plot for the blank solution was significantly smaller than those obtained for inhibitor-containing systems, indicating a lower apparent activation energy (*E*_a_ = 22.96 kJ mol^−1^) for the uninhibited corrosion process. In marked contrast, the addition of all four imidazoline derivatives substantially increased the activation energies (S4-C11 *E*_a_ = 34.60 kJ mol^−1^), providing direct evidence for their inhibitory effects on the corrosion mechanism. The measured activation energies followed the consistent ranking: S4-C11 > S4-C13 > S4-C9 > S4-C7, which shows excellent agreement with the performance hierarchy established through comprehensive inhibition efficiency testing.

**Fig. 8:**
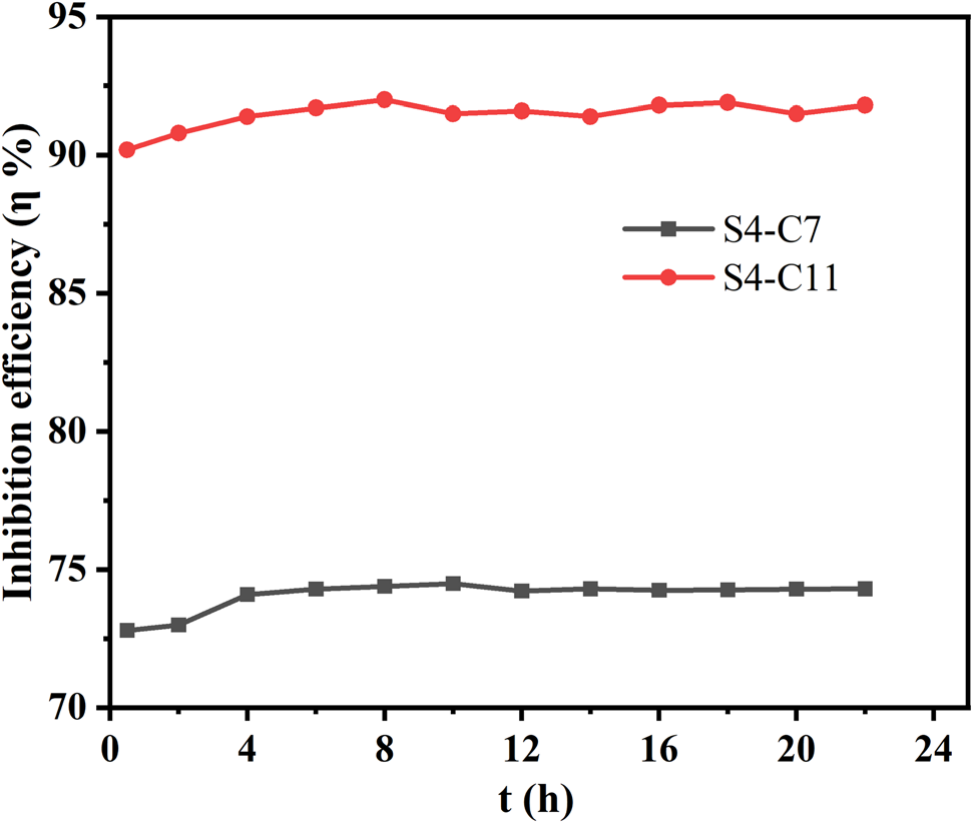
Time dependence of inhibition efficiency of S4-C11 and S4-C7 (100 mg mL^−1^) for Q235 carbon steel in CO_2_-saturated oilfield produced water at 90 °C.

Fig. 8 shows the stability of the corrosion inhibition performance of S4-C11 and S4-C7 at a concentration of 100 mg L^−1^, determined by measuring EIS every 2 hours. The results indicate that the corrosion inhibition rates of S4-C11 and S4-C7 show a gradual increase over the first 8 hours and remain relatively stable over the subsequent 14 hours. However, the corrosion inhibition rate of S4-C11 consistently remains above 90%, while that of S4-C7 stays above 73%. Both exhibit good stability, with S4-C11 demonstrating a higher corrosion inhibition rate.

### 3.7 Surface analysis

**Fig. 9:**
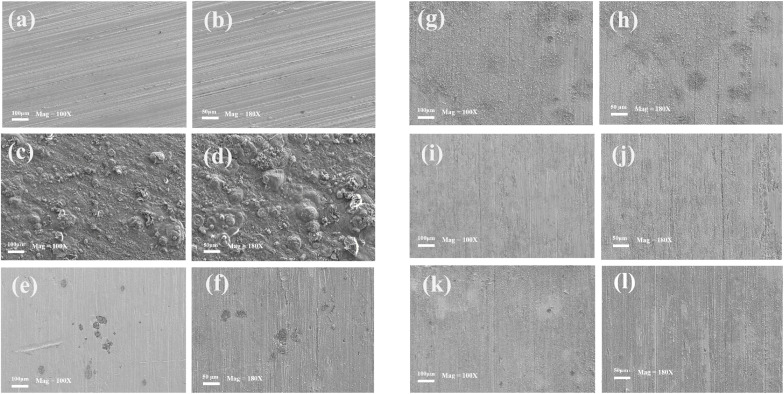
Surface morphology analysis of carbon steel after corrosion for 22 h at 90 °C in solution without or containing 100 mg per L imidazoline derivatives (SEM) (a and b) uncorroded carbon steel; (c and d) blank; (e and f) S4-C7; (g and h) S4-C9; (i and j) S4-C11; (k and l) S4-C13.

Fig. 9 shows the surface morphology of carbon steel specimens after 22 hours of corrosion at 90 °C in solutions without and with 100 mg per L imidazoline derivatives. The surface morphology analysis reveals distinct corrosion protection effects: the uncorroded carbon steel (Fig. 9(a) and (b)) displays a smooth surface with only mechanical polishing marks, while the blank solution-treated specimens (c and d) exhibit severely roughened surfaces. Specimens with S4-C7 (e and f) and S4-C9 (g and h) additives show minor corrosion traces but demonstrate noticeable protection compared to the blank, whereas those treated with S4-C11 (i and j) and S4-C13 (k and l) present significantly smoother, more uniform surfaces with minimal corrosion dam.

**Fig. 10:**
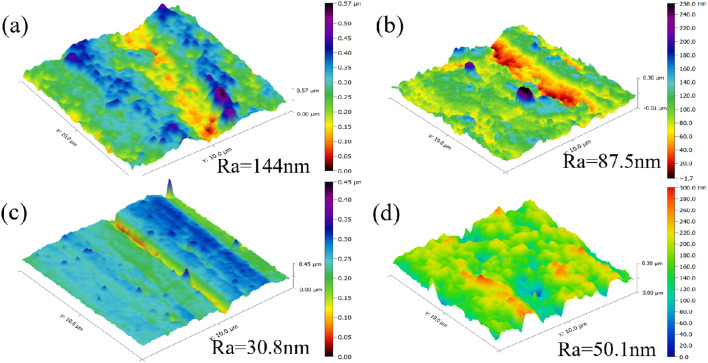
Surface roughness analysis of carbon steel after corrosion for 22 h in a solution without or containing 100 mg per L imidazoline derivatives at 90 °C (a) S4-C7; (b) S4-C9; (c) S4-C11; (d) S4-C13.

As shown in Fig. 10, quantitative atomic force microscopy (AFM) analysis revealed that carbon steel specimens corroded in blank solution exhibited an average surface roughness (*R*_a_) of 157 nm, whereas those treated with imidazoline derivatives showed markedly smoother surfaces with *R*_a_ values of 144 nm (S4-C7), 87.5 nm (S4-C9), 30.8 nm (S4-C11), and 50.1 nm (S4-C13), demonstrating an unambiguous inhibitor performance ranking of S4-C11 > S4-C13 > S4-C9 > S4-C7 in corrosion protection effectiveness.

**Fig. 11:**
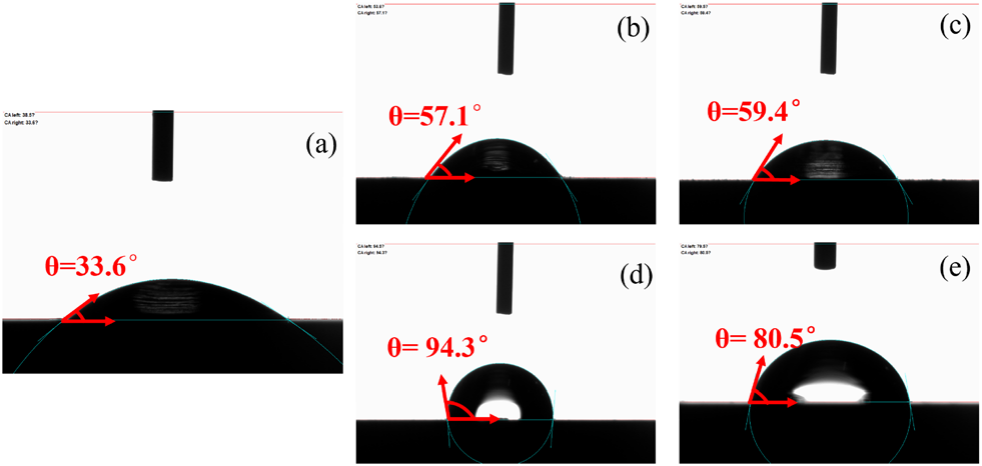
Surface contact angle test results of carbon steel soaked for 4 h in a solution without or containing 100 mg per L imidazoline derivatives at 90 °C (a) Blank; (b) S4-C7; (c) S4-C9; (d) S4-C11; (e) S4-C13.

The contact angle measurements of carbon steel specimens after 4 hours of immersion in solutions containing and lacking corrosion inhibitors are illustrated in Fig. 11. The blank solution sample, lacking protective inhibitor films, exhibited the smallest contact angle (33.6°), indicating complete surface exposure to corrosive species. In contrast, inhibitor-treated specimens showed significantly increased contact angles^39,40^ (up to 94.3°), demonstrating enhanced surface hydrophobicity from adsorbed inhibitor layers that effectively block electron transfer between corrosive particles and Fe substrates. The contact angle measurements for the four imidazoline derivatives followed the order: S4-C11 > S4-C13 > S4-C9 > S4-C7, directly correlating with their corrosion protection performance.

**Fig. 12:**
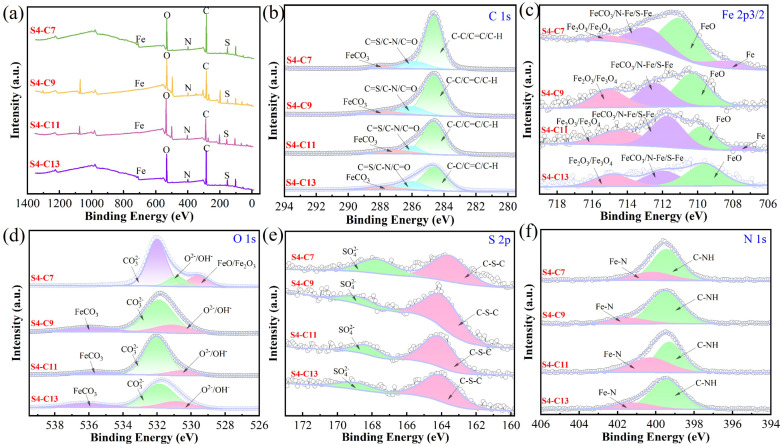
Surface XPS test results of carbon steel soaked in 100 mg per L corrosion inhibitor solution at 90 °C for 22 h (a) general spectrum (b) Fe 2p_3/2_ spectrum (c) O spectrum (d) C spectrum (e) S spectrum (f) N spectrum.

The surface composition of corroded carbon steel was characterized using XPS analysis, which also confirmed the adsorption of inhibitor molecules. Fig. 12 presents the XPS spectra obtained from carbon steel specimens after 22 hour immersion in inhibitor-containing solutions.

The survey XPS spectra (Fig. 12(a)) of all four imidazoline derivatives exhibited characteristic peaks of nitrogen (N) and sulfur (S), these findings substantiate the interfacial adsorption behavior of corrosion inhibitors on carbon steel substrates, thereby elucidating their corrosion inhibition mechanism. In the high-resolution C 1s spectrum (Fig. 12(b)), three distinct peaks were observed at binding energies of 284.60 eV, 285.96 eV, and 287.38 eV, corresponding to C–C/C=C/C–H, C=S/C–N/C=O,^41^ and FeCO_3_ species,^42^ respectively. Fig. 12(c) presents the high-resolution Fe 2p_3/2_ spectra for all four inhibitor-treated samples, showing three characteristic peaks at binding energies of 709.14 eV (FeO), 711.21 eV (FeCO_3_/N–Fe/S–Fe), and 714.04 eV (Fe_2_O_3_/Fe_3_O_4_).^43^ Notably, the S4-C7 spectrum exhibited an additional metallic Fe peak (706.7 eV).^44^ This observation can be attributed to insufficient corrosion duration leaving residual uncorroded Fe substrate exposed. Interestingly, the S4-C11 sample also showed a detectable metallic Fe signal. In this case, the presence of unoxidized Fe suggests that S4-C11 formed a more complete and effective inhibitor film that successfully protected the carbon steel substrate from corrosion. This interpretation is further supported by complementary weight loss measurements, electrochemical data, and SEM characterization results. The O 1s spectra (Fig. 12(d)) for S4-C9, S4-C11, and S4-C13 inhibitors exhibited three characteristic peaks at binding energies of 530.54 eV (O^2−^/OH^−^),^45^ 532.07 eV (CO_3_^2−^),^46^ and 535.67 eV (FeCO_3_). In contrast, the S4-C7 inhibitor showed a distinct peak at 529.60 eV corresponding to FeO/Fe_2_O_3_ instead of FeCO_3_. This difference suggests that the short carbon chain in S4-C7 may lead to incomplete surface adsorption. The S 2p spectra (Fig. 12(e)) revealed two characteristic peaks at 164.13 eV (C–S–C) and 168.29 eV (SO_4_^2−^),^42^ demonstrating both the intact inhibitor structure and partial oxidation products. The N 1s spectra (Fig. 12(f)) displayed two distinct peaks at 399.33 eV (C–NH) and 400.3 eV (N–Fe),^47^ confirming the formation of inhibitor-surface coordination bonds.

### 3.8 Quantum chemical calculations

**Fig. 13:**
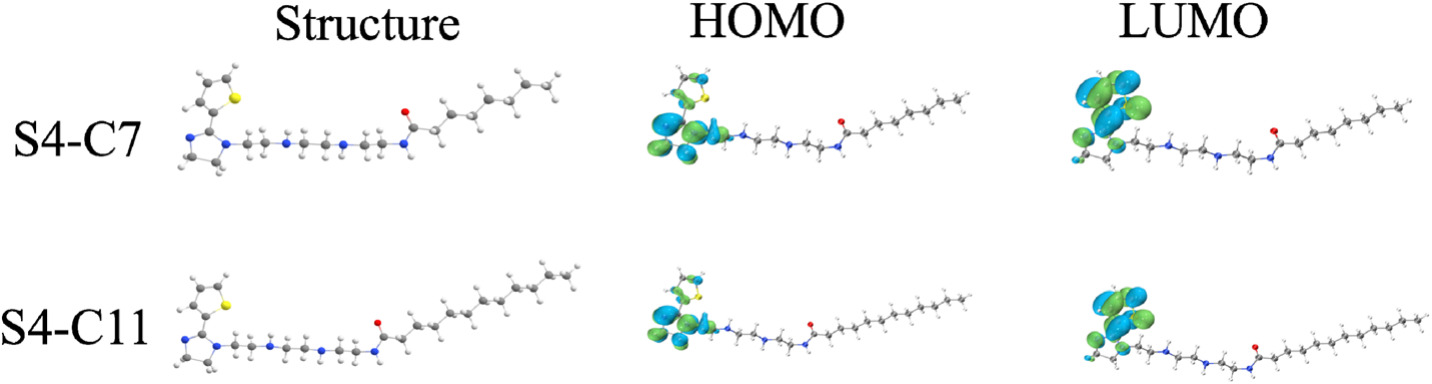
Optimized structure and HOMO and LUMO front track of S4-C7 and S4-C11.

The optimized molecular structures of S4-C7 and S4-C11, along with their corresponding distributions of highest occupied molecular orbital (HOMO) and lowest unoccupied molecular orbital (LUMO), were computationally determined. These findings are graphically represented in Fig. 13. For both S4-C7 and S4-C11, the highest occupied molecular orbitals (HOMO) are predominantly concentrated on the nitrogen atoms of the imidazoline ring and the sulfur atoms of the thiophene ring. This localization pattern suggests that these specific atoms have a high propensity to donate their lone-pair electrons, thereby facilitating the formation of coordinate bonds with the vacant orbitals of iron. In contrast, the LUMO orbitals are predominantly distributed across the entire thiophene ring.

**Fig. 14:**
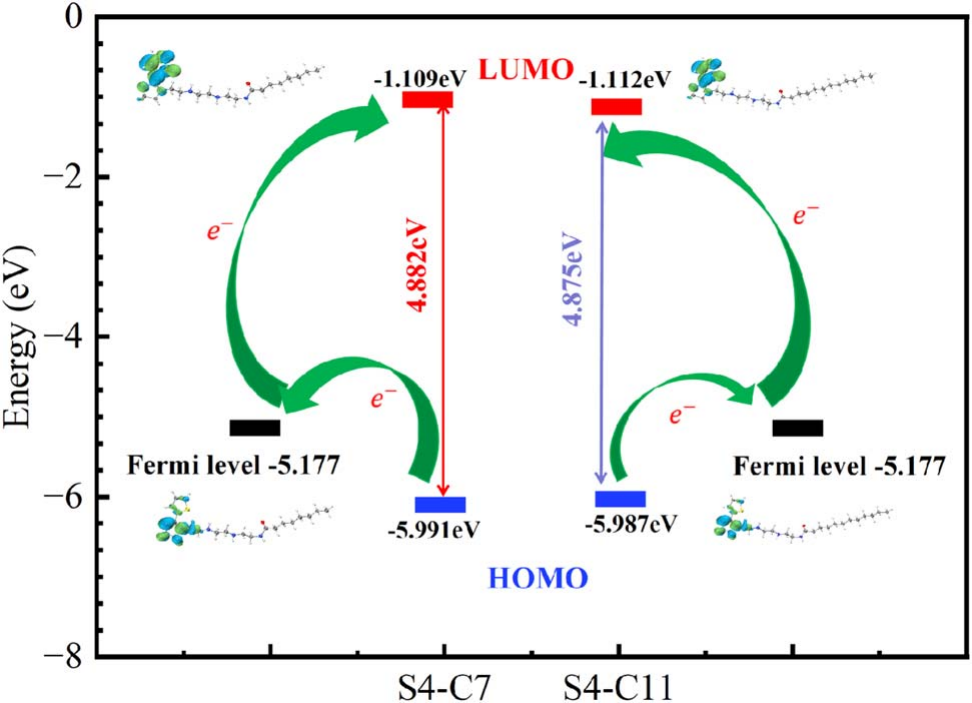
HOMO and LUMO orbital energies and their bandwidths Δ*E* of S4-C7 and S4-C11 molecules.

As depicted in Fig. 14, the orbital energies of the highest occupied molecular orbital (HOMO) and the lowest unoccupied molecular orbital (LUMO), along with the respective energy gaps calculated as Δ*E* = *E*_LUMO_ − *E*_HOMO_, are shown for S4-C7 and S4-C11. The highest occupied molecular orbital (HOMO) and the lowest unoccupied molecular orbital (LUMO) are indicative of a molecule's electron-donating and electron-accepting abilities, respectively. The energy gap Δ*E*, defined as the difference between the energy of the LUMO and the HOMO, is a pivotal parameter for quantifying and evaluating these electronic properties. A smaller Δ*E* value corresponds to stronger electron-donating ability, facilitating covalent bond formation with vacant orbitals of transition metals and consequently enhancing inhibitor adsorption on metal surfaces.^48^ Comparative analysis reveals that S4-C11 (Δ*E* = 4.875 eV) possesses a narrower energy gap than S4-C7 (Δ*E* = 4.882 eV), indicating superior electron-donating capacity of S4-C11, which correlates precisely with its higher corrosion inhibition performance (S4-C11 > S4-C7) as demonstrated experimentally.

### 3.9 Molecular dynamics (MD) simulations

**Fig. 15:**
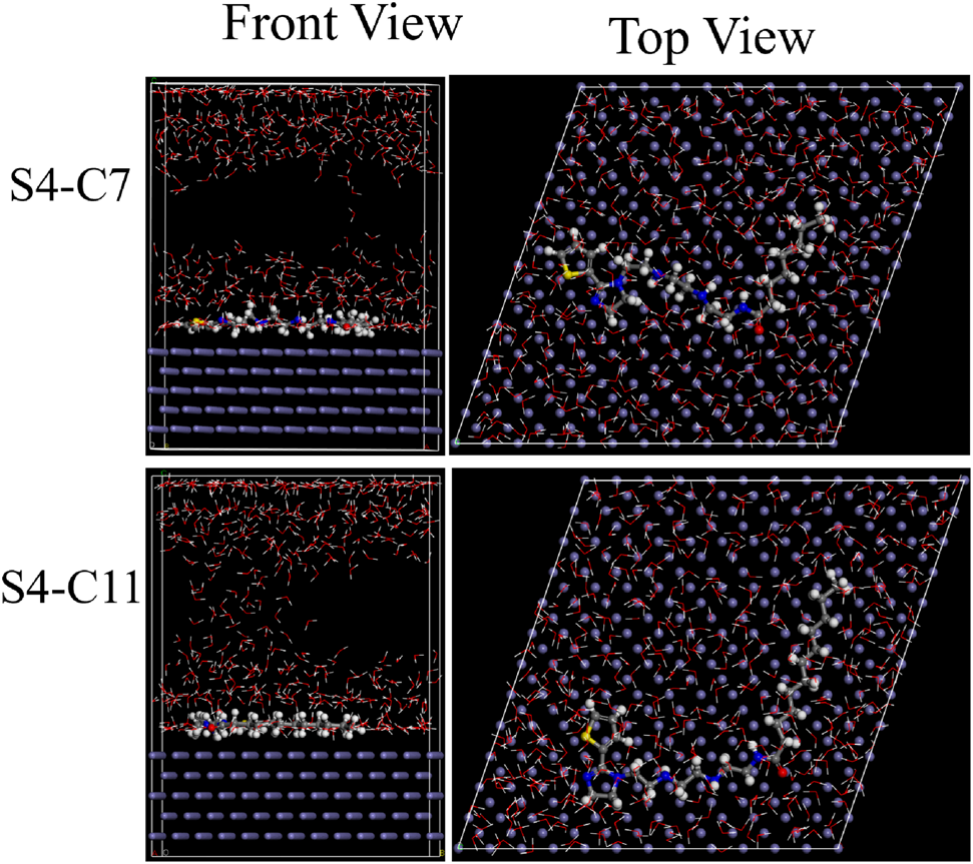
Molecular kinetic adsorption model of S4-C7 and S4-C11 on Fe (110) surface.

In order to elucidate the interaction mechanisms between the carbon steel interfaces and the S4-C7/S4-C11 corrosion inhibitors, molecular dynamics simulations were carried out. Fig. 15 displays the optimal adsorption configurations of S4-C7 and S4-C11 inhibitors on carbon steel surfaces, obtained through the adsorption locator module. The two inhibitor molecules, S4-C7 and S4-C11, adsorb in a parallel orientation onto the Fe (110) surface, achieving the highest possible surface coverage. The adsorption energy (*E*_ads_ = *E*_ttel_ − (*E*_inh_ + *E*_surf_)) was calculated to be −232.50 kcal mol^−1^ for S4-C7 and −289.82 kcal mol^−1^ for S4-C11. The larger absolute value of *E*_ads_ for S4-C11 indicates stronger adsorption capability compared to S4-C7, resulting in denser film formation and superior corrosion inhibition performance.^30^

**Fig. 16:**
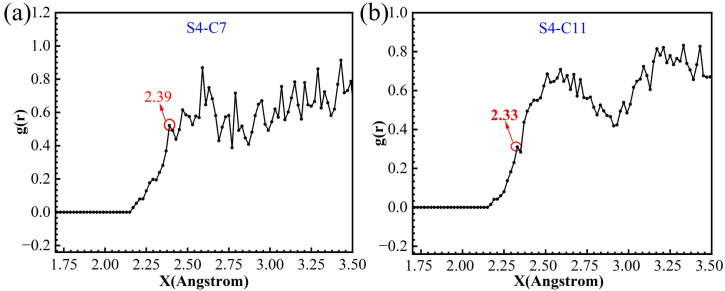
Radial distribution function of S4-C7 (a) and S4-C11 (b) adsorbed on Fe (110) surface.

The radial distribution function (RDF, *g*(*r*)) obtained from molecular dynamics simulations was employed to analyze the distance between adsorbed inhibitor molecules and the Fe surface. Generally, the emergence of the first peak in the radial distribution function (RDF) profile within the 1–3.5 Å interval serves as an indicator of chemical adsorption occurring on the carbon steel surface. As illustrated in Fig. 16, the first peaks of the radial distribution functions for S4-C7 (2.39 Å) and S4-C11 (2.33 Å) are located below 3.5 Å. This observation provides conclusive evidence for the occurrence of chemical adsorption on the carbon steel surface, a result that aligns well with the outcomes of thermodynamic calculation. Furthermore, the shorter Fe-surface distance observed for S4-C11 (2.33 Å *vs.* 2.39 Å for S4-C7) demonstrates its stronger adsorption capability on carbon steel.

**Fig. 17:**
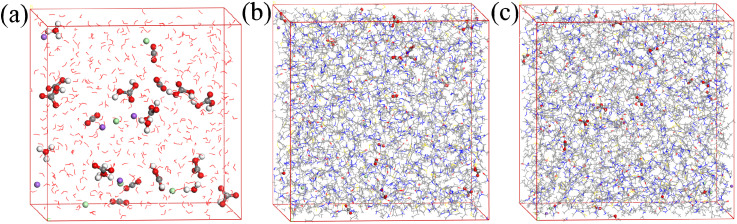
Diffusion models of Cl^−^, H_3_O^+^, H_2_O, CO_2_, HCO_3_^−^, H_2_CO_3_ in blank water phase and corrosion inhibitor film phase (a) H_2_O phase; (b) S4-C7 phase; (c) S4-C11 phase.

Molecular dynamics (MD) simulations were conducted to explore the diffusion behavior of corrosive species (H_2_O, H_3_O^+^, HCO_3_^−^, H_2_CO_3_, CO_2_, Cl^−^) within both the aqueous phase and the inhibitor film phase. The primary objective of this investigation was to ascertain the diffusion coefficients (*D*) of these species, which are crucial parameters for a assessment of the corrosion inhibition performance. As depicted in Fig. 17, the diffusion models of the corrosive particles within the aqueous phase and the inhibitor film phase are illustrated.

The diffusion coefficients (*D*) of different corrosive species were calculated through the process of fitting the slopes of their respective mean square displacement curves. The resultant data and corresponding findings are systematically tabulated in Table 4. The data reveal significantly lower diffusion coefficients in both S4-C7 and S4-C11 inhibitor films compared to the blank solution-exemplified by H_3_O^+^ (decreasing from 0.291971 m^2^ s^−1^ to 0.001480 m^2^ s^−1^ in S4-C11 film) and Cl^−^ (reduced from 1.101241 m^2^ s^−1^ to 6.33 × 10^−5^ m^2^ s^−1^ in S4-C11 film). This marked reduction demonstrates that the adsorbed inhibitor films effectively obstruct the diffusion of corrosive species toward the carbon steel surface, thereby exhibiting superior corrosion inhibition performance.

**Table 4:** Diffusion coefficients (m^2^ s^−1^) of different corrosive substances in aqueous solution (H_2_O) and adsorbed corrosion inhibitor film phases (S4-C7 and S4-C11)

Models	CO_2_	H_2_CO_3_	H_2_O	H_3_O^+^	HCO_3_^−^	Na^+^	Cl^−^
H_2_O box	0.217706	0.134796	0.286924	0.291971	0.331342	0.494781	0.101241
S4-C7 box	0.036814	0.001804	0.002447	0.001549	9.92 × 10^−4^	4.64 × 10^−4^	6.62 × 10^−4^
S4-C11 box	0.030888	0.001556	0.002336	0.001480	1.02 × 10^−4^	6.74 × 10^−4^	6.33 × 10^−5^

**Fig. 18:**
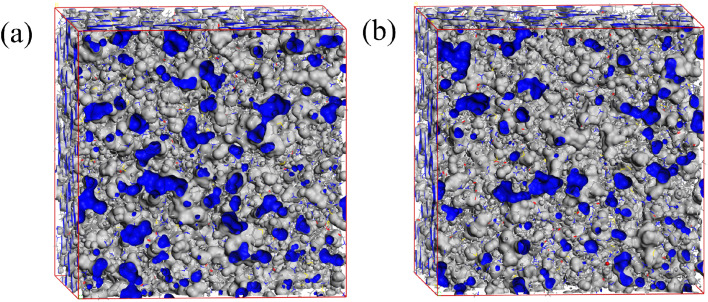
Diffusion models of Cl^−^, H_3_O^+^, H_2_O, CO_2_, HCO_3_^−^, H_2_CO_3_ in blank water phase and corrosion inhibitor film phase (a)S4-C7 phase; (b) S4-C11 phase.

Moreover, the free volume fraction (FFV), which is derived from the diffusion models of corrosive species in both the aqueous phase and the inhibitor phase, offers supplementary metrics for the assessment of corrosion inhibition performance.^49^ Typically, larger cavity volumes within the inhibitor film correspond to higher FFV values and consequently lower inhibition efficiency. Fig. 18 illustrates the distribution of free volume within the fabricated inhibitor films. The free volume (*V*_f_) and occupied volume (*V*_0_) of the inhibitor films were obtained through simulation, enabling calculation of the FFV according to the formula (10), with all results summarized in Table 5.10
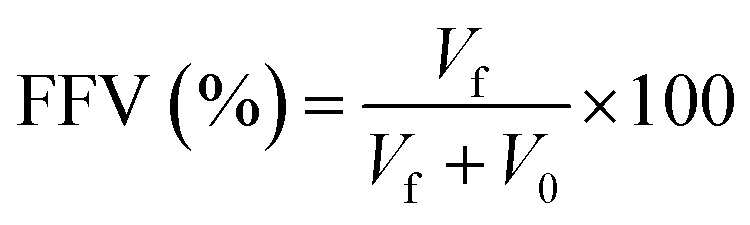


**Table 5:** Simulation results of free volume fraction of corrosion inhibitor film

System	FFV (%)	*V*_f_ (Å^3^)	*V*_0_ (Å^3^)
S4-C7	20.9	27 411.94	103 725.5
S4-C11	17.5	27 168.2	128 079.7

As evidenced by the data in Table 5, the free volume fraction (FFV) within the S4-C11 inhibitor film (17.5%) is significantly lower than that of the S4-C7 film (20.9%), demonstrating that the S4-C11 film possesses a more compact molecular packing structure. This reduced FFV directly correlates with superior diffusion barrier properties, as the denser S4-C11 film more effectively impedes the penetration of corrosive species to the carbon steel surface compared to S4-C7, thereby exhibiting enhanced corrosion inhibition performance – a finding that shows excellent agreement with experimental measurements.

### 3.10 Corrosion inhibition mechanism

**Fig. 19:**
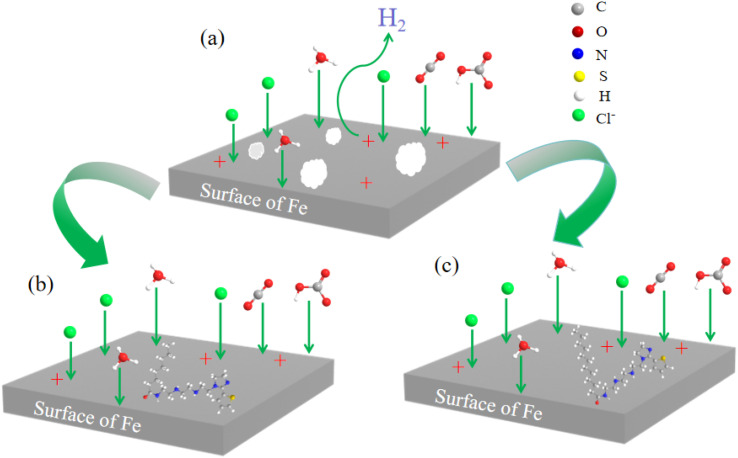
Corrosion inhibition mechanism of corrosion inhibitor in CO_2_ environment (a) blank (b) S4-C7 (c) S4-C11.

As shown in Fig. 19, in the blank system devoid of inhibitors (depicted in Fig. 19(a)), there is substantial diffusion of corrosive species, including H_2_O, H_3_O^+^, HCO_3_^−^, H_2_CO_3_, CO_2_, Cl^−^, towards the carbon steel surface. This diffusion process leads to pronounced corrosion damage on the carbon steel. Conversely, in the systems incorporating S4-C7 or S4-C11 molecules (as illustrated in Fig. 19(b) and (c)), the carbon steel/solution interface witnesses the development of protective molecular adsorption layers. These layers are formed *via* the combined action of physical and chemical adsorption mechanisms, which act in a synergistic manner. These highly organized interfacial films efficiently obstruct the diffusion routes of corrosive particles, thereby preventing their approach to the metal surface and impeding the corrosion process.

The chemical adsorption of S4-C7 and S4-C11 molecules onto carbon steel surfaces is primarily mediated by the formation of coordination bonds, specifically N–Fe and S–Fe bonds, through their sulfur- and nitrogen-containing functional groups. This interfacial bonding facilitates the self-assembly of inhibitor molecules into densely packed, well-ordered adsorption layers on the metal substrate. The generated films efficiently impede the diffusion of corrosive species towards the metal/solution interface, thereby conferring substantial corrosion protection. Notably, both density functional theory (DFT) calculations and electrochemical impedance spectroscopy (EIS) measurements consistently demonstrate that S4-C11 exhibits lower adsorption energy (Δ*E*_ads_) and higher charge transfer efficiency compared to S4-C7 characteristics that show strong positive correlation with its superior inhibition performance.

## 4. Conclusions

In the present investigation, four imidazoline derivatives, namely S4-C7, S4-C9, S4-C11, and S4-C13, differing in carbon chain lengths, were synthesized. These derivatives were developed as high-performance corrosion inhibitors for carbon steel in CO_2_-saturated oilfield produced water. Weight loss measurements and electrochemical analyses revealed that among the synthesized imidazoline derivatives, S4-C11 demonstrated exceptional corrosion inhibition efficacy. Specifically, at a low concentration of 100 mg L^−1^, S4-C11 achieved a corrosion inhibition efficiency of 87.55%, outperforming other tested compounds.

Both electrochemical experiments and theoretical calculations demonstrated that S4-C11 exhibited superior inhibition efficiency compared to S4-C7. The extended carbon chain length in S4-C11 enhanced its inhibitory performance through two key mechanisms: (1) suppressing the diffusion volume of corrosive species (*e.g.*, Cl^−^, H^+^) toward the carbon steel surface, and (2) strengthening hydrophobicity *via* the formation of a densely packed molecular structure. This modified imidazoline derivative provides a theoretical foundation for designing high-performance inhibitors tailored to moderate-to-high temperature environments, thereby advancing the development of next-generation imidazoline-based corrosion inhibitors.

## Conflicts of interest

There are no conflicts to declare.

## Supplementary Material

RA-015-D5RA04201A-s001

## Data Availability

Data will be made available on request. Supplementary information is available. See DOI: https://doi.org/10.1039/d5ra04201a.
