# Supplementary material for: Thiophene-imidazoline derivatives with varying chain lengths modified by organic acids as corrosion inhibitors for carbon steel in the CO2-saturated oilfield produced water
Source: RSC Adv. 2025 Sep 3;15(38):31632–50. doi: 10.1039/d5ra04201a (PMC12406301; doi:10.1039/d5ra04201a)
Supplement: RA-015-D5RA04201A-s001 [file RA-015-D5RA04201A-s001.pdf]

Supplementary Information (SI) for RSC Advances.
This journal is © The Royal Society of Chemistry 2025

Thiophene-imidazoline derivatives with varying chain lengths modified by Organic Acids as corrosion inhibitors for carbon steel in the CO_2-saturated oilfield produced water

Shuxin Jia [a], Lei Xiong [a], Sisi Du [a], Lin Shen [a], Yonggang Yu [b], Jiangbing Li [a,*], Zhenglei Wu [c,*]

[a] School of Chemistry and Chemical Engineering/State Key Laboratory Incubation Base for Green Processing of Chemical Engineering, Shihezi University, Shihezi 832003, China. E-mail: jiashuxin2021@163.com (Shuxin Jia)，18891556110@163.com (Lei Xiong), dusisi@stu.shzu.edu.cn (Sisi Du), 18841518147@163.com (Lin Shen) ljbing@126.com (Jiangbing Li)

[b] Karamay Zhongke Hengxin Technology Co., Ltd, Karamay, China. Email：jingsenyu@126.com (Yonggang Yu)

[c] Xinjiang Jintai Advanced Material Technologies Co. Ltd, Huyanghe, China. Email：13912944872@139.com (Zhenglei Wu)

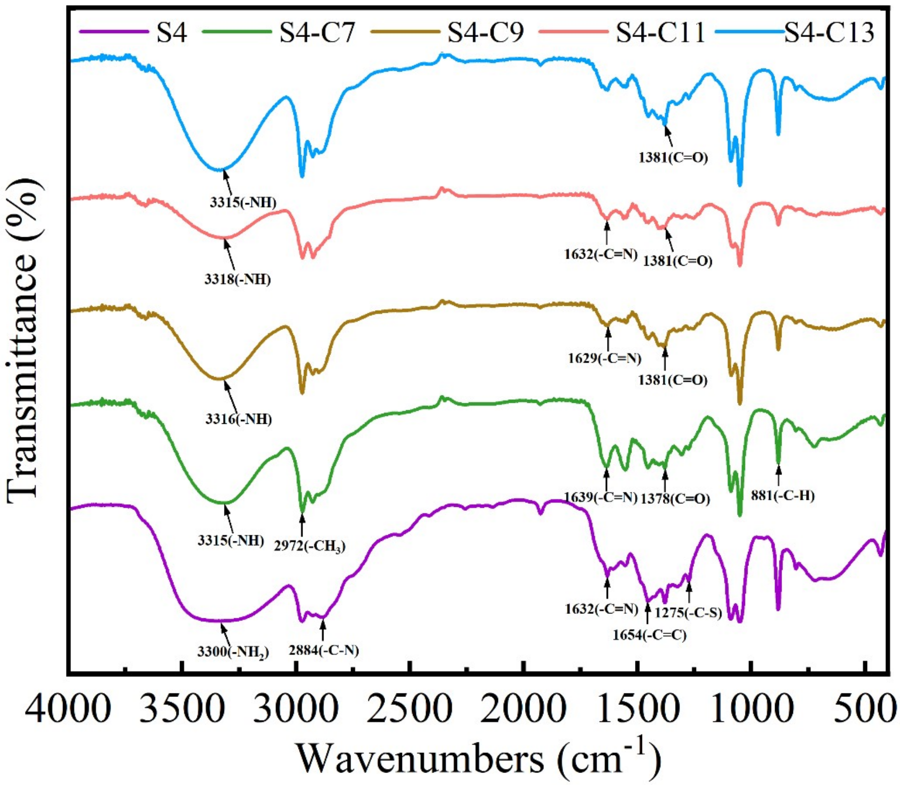

Figure S1. FT-IR spectra of the imidazoline derivative (S4-C7 to S4-C13)

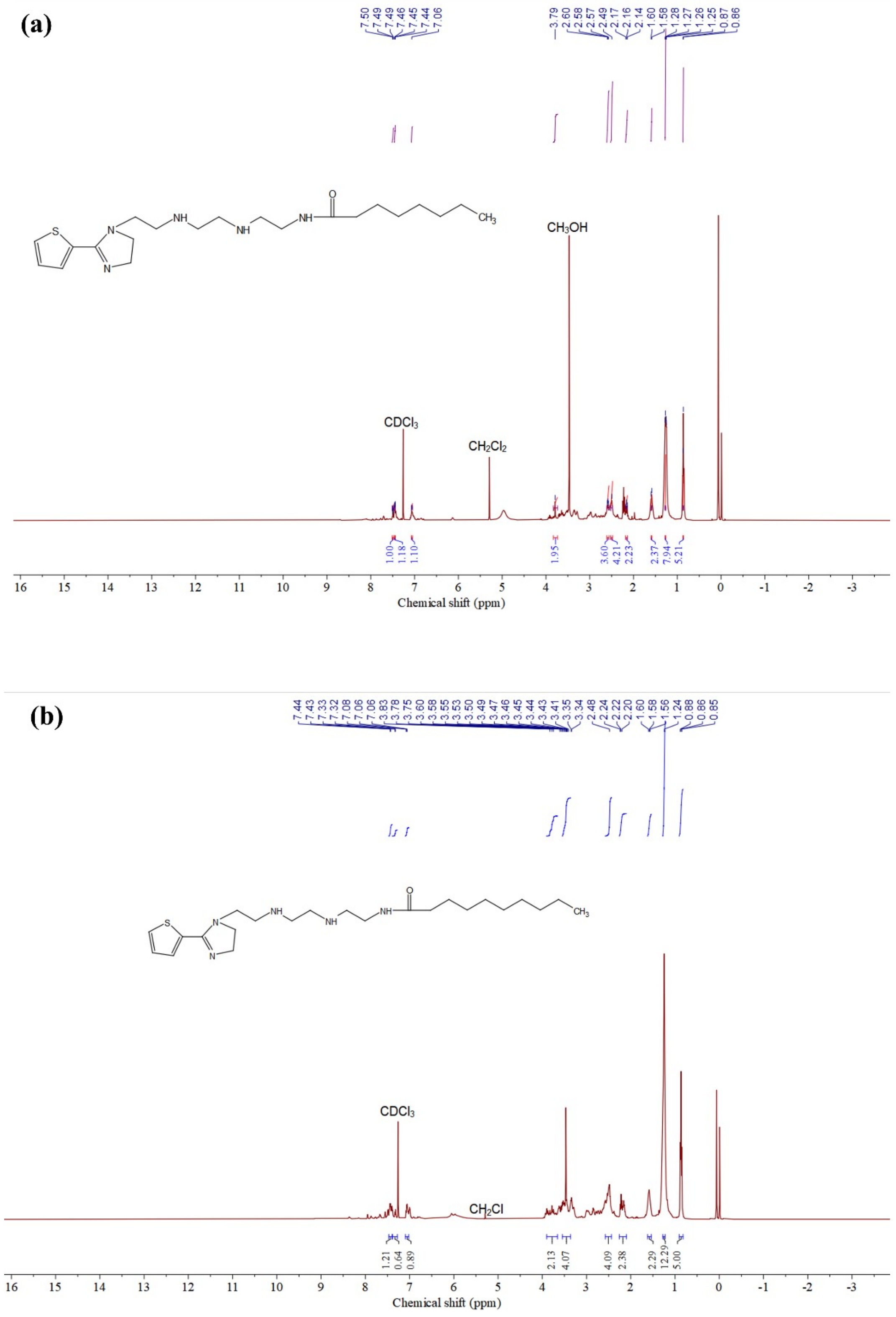
(a)
7.50 7.49 7.49 7.46 7.45 7.44 7.06
3.79 2.60 2.58 2.57 2.49 2.17 2.16 2.14 1.60 1.58 1.28 1.27 1.26 1.25 0.87 0.86
S
N
N
NH
NH
NH
O
CH3
CH3OH
CDCl3
CH2Cl2
1.00 1.18 1.10
1.95
3.60 4.21 2.23
2.37 7.94 5.21
16 15 14 13 12 11 10 9 8 7 6 5 4 3 2 1 0 -1 -2 -3
Chemical shift (ppm)
(b)
7.44 7.43 7.33 7.32 7.08 7.06 7.06 3.83 3.78 3.75 3.60 3.58 3.55 3.53 3.50 3.49 3.47 3.46 3.45 3.44 3.43 3.41 3.35 3.34 2.48 2.24 2.22 2.20 1.60 1.58 1.56 1.24 0.88 0.86 0.85
CDCl3
CH2Cl
1.21 0.64 0.89
2.13 4.07
4.09 2.38
2.29 12.29 5.00
16 15 14 13 12 11 10 9 8 7 6 5 4 3 2 1 0 -1 -2 -3
Chemical shift (ppm)

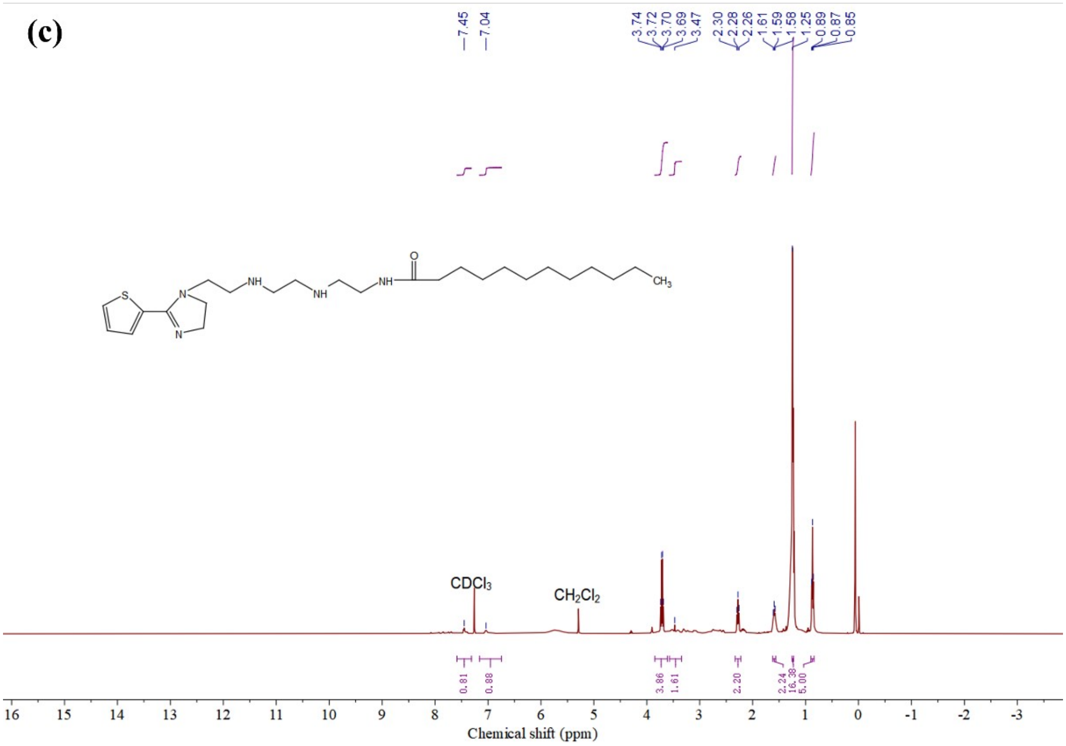

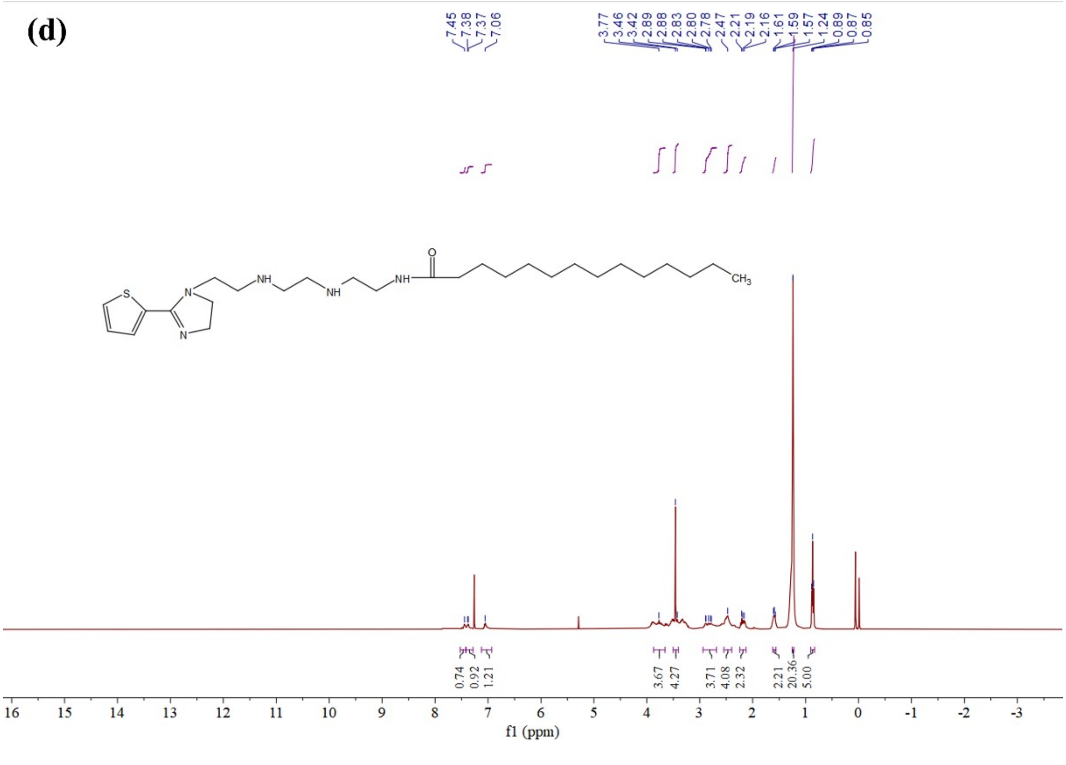

Fig S2. ^{1}H NMR spectroscopy of imidazoline derivatives: (a) S4-C7 (b) S4-C9 (b) S4-C11 (b) S4-C13.

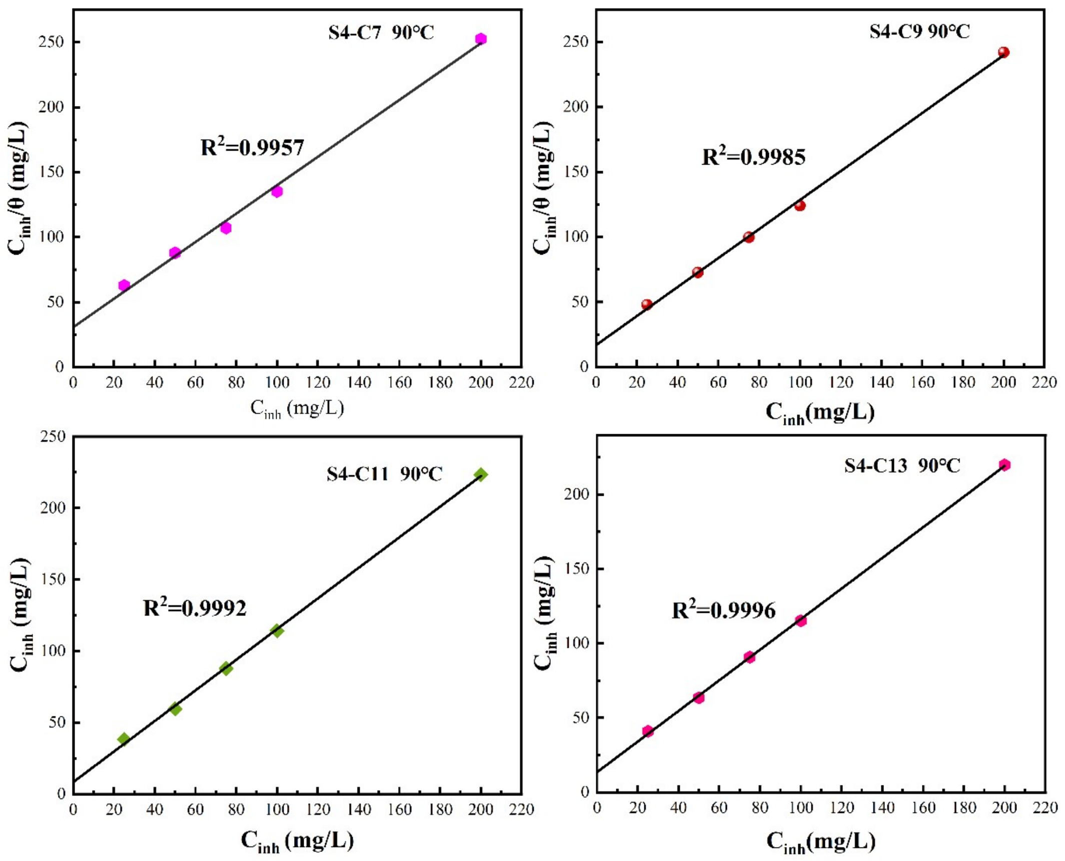

Figure S3 Langmuir adsorption isotherms of four imidazoline derivatives at 90℃

Table S1 Thermodynamic parameters for the adsorption of four imidazoline derivatives on carbon steel surface

inhibitor	T（K）	Slope	Intercept	K_{ads}	ΔG^0_{ads} ($KJ•mol^{-1}$)
S4-C7	303	1.0734	2.7417	0.3647	-32.28
	323	1.0922	7.3732	0.1356	-31.75
	343	1.0651	20.6100	0.0485	-30.78
	363	1.0920	30.7633	0.0325	-31.35
S4-C9	303	1.0595	1.9816	0.5048	-33.10
	323	1.0495	4.7586	0.2101	-32.93
	343	1.0867	7.7426	0.1292	-33.58
	363	1.1150	16.8954	0.0592	-33.18
S4-C11	303	1.0588	0.3464	2.8870	-37.49
	323	1.0519	1.9430	0.7603	-36.38

Continued Table 4

S4-C11	343	1.0632	1.9022	0.3374	-34.82
S4-C11	363	1.0704	8.3137	0.1203	-35.32
S4-C13	303	1.0713	0.4469	2.2375	-36.85
	323	1.0611	2.5551	0.5535	-35.53
S4-C13	343	1.0898	1.7417	0.5741	-37.83
	363	1.0278	10.5514	0.0996	-34.75

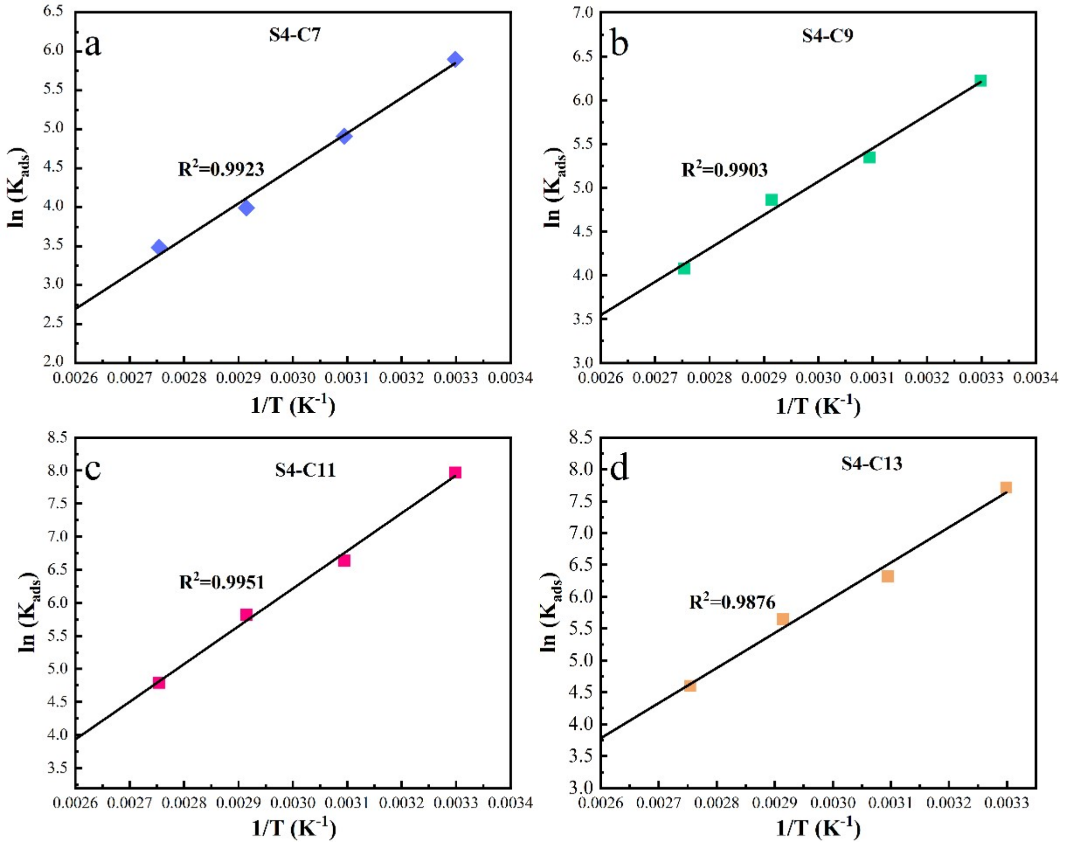

Figure S4 Relationship of adsorption equilibrium constant with temperature

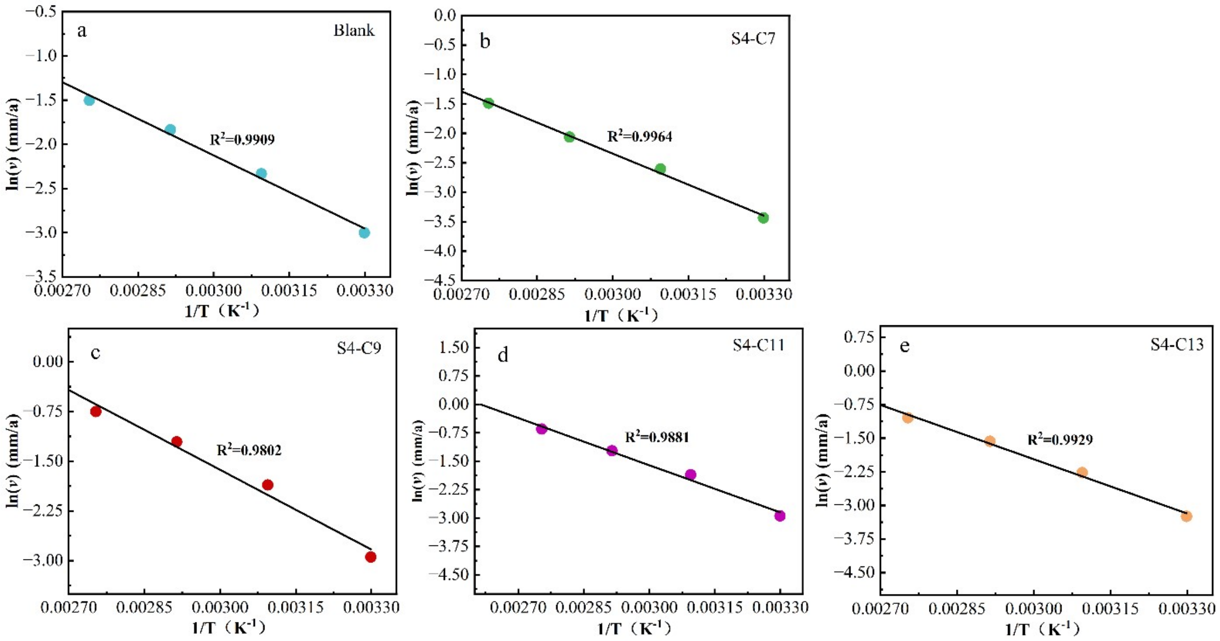

Figure S5 Arrhenius plot of corrosion rate for carbon steel

Table S2 Parameters of Arrhenius formula

Inhibitor	R^2	Slope	Ea（kJ/mol）
Blank	0.99094	-2761.51	22.96
S4-C7	0.99642	-3519.68	29.26
S4-C9	0.98017	-4012.69	33.36
S4-C11	0.98814	-4161.71	34.60
S4-C13	0.99288	-4037.92	33.43
